# Novel Techniques and Emerging Technologies in Chronic Total Occlusion Percutaneous Coronary Intervention: A Structured, Narrative Review

**DOI:** 10.3390/jpm16100538

**Published:** 2026-10-21

**Authors:** Michał Dobrzyński, Paweł Warzyszak, Bartosz Zięba

**Affiliations:** Cardiology Clinic, 1st Military Hospital, John Paul II Catholic University of Lublin, 20-049 Lublin, Poland; pawel.warzyszak@gmail.com (P.W.); ziebabartosz@gmail.com (B.Z.)

**Keywords:** chronic total occlusion, CTO PCI, hybrid approach, intravascular imaging, calcium modification, drug-coated balloons, novel CTO technologies

## Abstract

Chronic total occlusions (CTOs) represent one of the most technically demanding areas of percutaneous coronary intervention (PCI). Despite advances in equipment and operator expertise, CTO PCI remains associated with lower procedural success rates and a higher risk of complications than non-CTO PCI. This review summarizes emerging techniques and technologies designed to improve procedural efficiency, safety, and outcomes in contemporary, personalized patient care. Recent innovations span multiple domains of CTO PCI. The minimalistic hybrid approach (MHA) aims to reduce vascular complications by minimizing the number of access sites and the sheath size. Novel perforation management strategies, including the cut balloon technique (CBT) and the condom technique, offer simple, low-cost options for distal vessel sealing, supported so far only by small series and technical reports. Advanced re-entry methods, such as tip-detection, antegrade dissection and re-entry (TD-ADR), integrate intravascular ultrasound (IVUS) to enhance precision. HydroDynamic contrast recanalization (HDR) revisits hydraulic dissection with a controlled, microcatheter-based contrast strategy whose safety profile remains to be established. The microcatheter anchor technique (MAT) has been proposed to facilitate retrograde CTO PCI in post-CABG anatomy. New plaque-modifying strategies, including RODIN-CUT and ORCAS, leverage high-pressure cutting balloon inflations under real-time IVUS guidance. Additional technologies, such as Plasmawire™, Shockwave Javelin^®^, and Hertz Contact intravascular lithotripsy (IVL), may expand the armamentarium for crossing and modifying complex CTO lesions, although CTO-specific clinical data are very limited (for Javelin, a 20-patient first-in-human coronary series). CT coronary angiography (CTCA) continues to improve pre-procedural planning, while drug-coated balloons (DCBs) show promise in selected de novo CTO lesions, although the evidence remains limited to retrospective series. Taken together, this rapid pace of innovation is steadily widening the procedural toolkit available for CTO PCI and may improve outcomes. Evidence for most of these techniques comes from case reports, small single-center series, and early-phase single-arm studies. It suggests feasibility in selected patients, but safety data are limited, and some reports signal concerns, including tamponade after the cut balloon technique and a higher perforation rate in a registry of the Carlino technique. The place of these techniques in routine practice will become clear only once larger, multicenter trials are completed.

## 1. Introduction

Chronic total occlusions (CTOs) are among the most challenging coronary interventions and are associated with a relatively high complication rate. They often require costly dedicated equipment and highly trained, experienced operators. Although symptom relief is frequently observed in patients with a high ischemic burden, randomized trials have shown that CTO PCI improves angina-related health status in symptomatic patients (EuroCTO) [1], whereas no reduction in a composite of death, myocardial infarction, stroke, or revascularization was demonstrated in DECISION-CTO [2]; both trials were stopped before reaching their planned enrollment. Registry data suggest that CTO PCI may be considered when it is expected to improve left ventricular ejection fraction in patients with heart failure with mildly reduced or reduced ejection fraction (HFmrEF or HFrEF) [3,4], to reduce a large ischemic burden, or to help manage persistent ventricular arrhythmias; these indications rest on observational evidence.

Because CTO lesions vary substantially, operators should be familiar with all established crossing strategies to maximize procedural efficiency. Depending on the crossing site and guidewire position, antegrade wiring (AW), retrograde wiring (RW), antegrade dissection and re-entry (ADR), or retrograde dissection and re-entry (RDR) may be used. As CTO techniques and equipment continue to develop rapidly, continuous training is essential. Technical success in CTO PCI has reached a high level in experienced centers. The international PROGRESS-CTO registry, encompassing more than 10,000 procedures performed at 40 centers between 2012 and 2022, showed an increase in technical success from 81.6% in 2016 to 88.1% in 2021 [5], without a corresponding increase in major adverse cardiovascular events. Similar results, in the range of 85–90%, have been confirmed by other large international registries, including the European ERCTO registry (8673 procedures performed in 2021–2022), in which the overall technical success rate was 89.1%, and the rate of major adverse cardiac and cerebrovascular events was 1.7% [6,7]. This review focuses on recently emerging strategies and technologies in CTO PCI, with emphasis on procedural rationale, available clinical evidence, technical limitations, and potential integration into contemporary practice. It covers techniques and technologies used in CTO PCI, including those that are only now entering this field.

## 2. Methods

### 2.1. Design

This is a structured narrative review, prepared with reference to the SANRA quality criteria for narrative reviews. No formal meta-analysis or risk-of-bias assessment was planned.

### 2.2. Information Sources and Dates

PubMed/MEDLINE, Embase, Scopus, and Web of Science were searched from January 2011 to October 2026. ClinicalTrials.gov was searched for ongoing coronary studies of emerging devices. Conference abstracts were included only when indexed in these databases (for example, in journal supplements of major interventional meetings) and are labeled as such. For devices without peer-reviewed publications, manufacturer and trade-press sources were identified through targeted web searches. UpToDate and SpringerLink were used only for background and terminology verification, not as search databases. Reference lists of key articles were screened manually. Because this is a narrative review, no protocol was registered.

### 2.3. Search Strategy

Searches combined a core term set (“chronic total occlusion” OR CTO) AND (“percutaneous coronary intervention” OR PCI) with topic-specific terms, each searched separately and adapted to the indexing of each database:

- Access and algorithms: “minimalistic hybrid approach”, “hybrid algorithm”, “radial access”.
- Perforation: “cut balloon technique”, “balloon fragment”, “condom technique”, “coronary perforation” AND “sealing”.
- Crossing and re-entry: “tip detection”, “antegrade dissection and re-entry”, Stingray, “Carlino technique”, “HydroDynamic contrast recanalization”, “microcatheter anchor”, Plasmawire, “radiofrequency wire”.
- Calcium and lesion preparation: “RODIN-CUT”, ORCAS, “cutting balloon” AND IVUS, “intravascular lithotripsy”, Javelin, “Hertz contact”, LithiX.
- Imaging and adjuncts: “CT-RECTOR”, “coronary CT angiography” AND CTO, “optical coherence tomography” AND CTO, “drug-coated balloon” AND (CTO OR “in-stent restenosis”).

### 2.4. Eligibility Criteria

Inclusion criteria were as follows:

- English-language randomized trials, observational studies, registries, consensus documents, reviews, and technical reports focused on CTO PCI techniques and technologies;
- For devices without peer-reviewed clinical data: conference abstracts, regulatory or manufacturer documents, and trade-press reports, explicitly labeled as such in the text and in the summary table in the Conclusions section;
- Case reports describing a technical novelty.

Exclusion criteria were as follows:

- Non-English publications;
- Case reports without technical novelty;
- Studies unrelated to coronary intervention.

Low-level sources were not used to support efficacy or safety claims.

### 2.5. Study Selection and Data Extraction

Two reviewers independently screened titles and abstracts and then assessed full texts; disagreements were resolved by consensus. The extracted data included the mechanism of the technique or device, clinical rationale and indications, procedural success and safety outcomes, limitations, regulatory status (CE/FDA) where available, and the design and sample size of each source.

### 2.6. Classification of Evidence

Sources were assigned to descriptive evidence levels: Level 1, randomized trial or meta-analysis of randomized trials; Level 2, prospective study, multicenter registry, or meta-analysis of observational studies; Level 3, retrospective single- or multicenter series; Level 4, case report, technical report, or first-in-human series; and Level 5, expert opinion, conference abstract, or non-peer-reviewed material. These levels are used in the summary table in the Conclusions section and do not constitute a formal risk-of-bias assessment.

### 2.7. Authors’ Own Experience

Technical observations from the authors’ own in vitro (bench) testing and clinical practice at the Cardiology Clinic, 1st Military Hospital in Lublin are included as practical commentary, not as evidence; the illustrations accompanying the descriptions of CBT, tip-detection antegrade dissection and re-entry, and HydroDynamic contrast recanalization come from this material, as does the illustration of the vascular-sealing modification described in Section 5. Own-experience statements are marked as such in the text and figure legends.

## 3. The Minimalistic Hybrid Approach (MHA)

**Figure 1:**
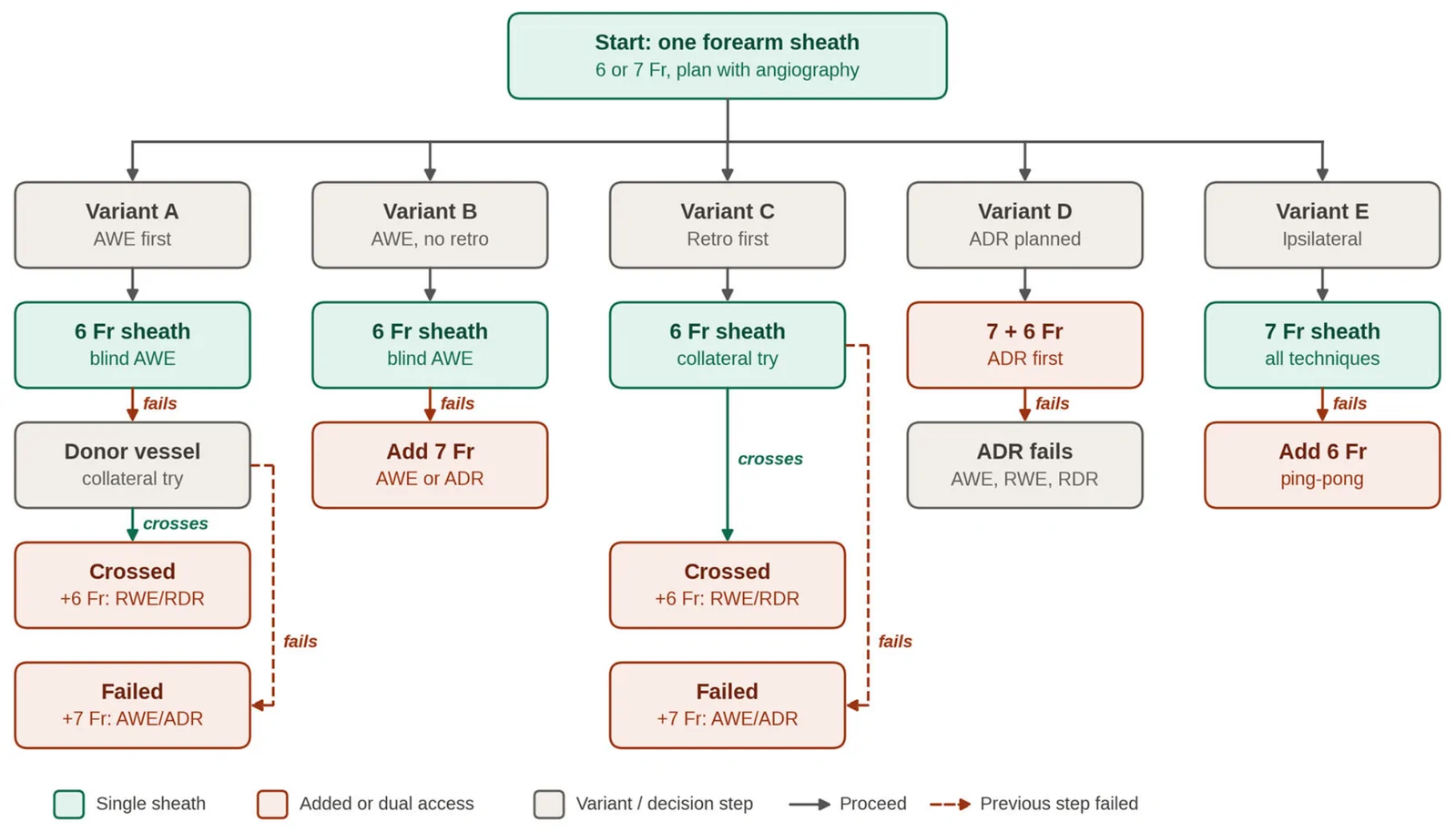
Algorithm of the minimalistic hybrid approach (MHA) for CTO PCI. All variants start with a single 6 or 7 Fr forearm sheath. Variants A and B begin with “blind” antegrade wire escalation (AWE) using a soft guidewire, in the presence (A) or absence (B) of contralateral interventional collaterals. Variant C begins with a retrograde attempt through contralateral collaterals. Variant D, planned for antegrade dissection and re-entry (ADR) in the absence of interventional collaterals, uses bilateral access (7 and 6 Fr) from the outset. Variant E applies when ipsilateral collaterals are the main source of visualization. Solid arrows indicate progression to the next step; dashed arrows indicate the next step if the previous one fails. Green boxes, single-sheath steps; red boxes, added or dual access. ADR, antegrade dissection and re-entry; AWE, antegrade wire escalation; RDR, retrograde dissection and re-entry; RWE, retrograde wire escalation.

Percutaneous recanalization of CTOs has traditionally required large-bore introducer sheaths and dual arterial access, with procedures often performed via the transfemoral route. This strategy has been associated with access-site complications, including bleeding, hematoma, pseudoaneurysm, arteriovenous fistula, and acute arterial closure. To reduce these risks, the minimalistic hybrid approach (MHA) has recently been proposed. MHA applies standard hybrid algorithm techniques while aiming to minimize the number of access sites, the catheter size, and the need for femoral access. It emphasizes careful pre-procedural anatomical assessment to select the most appropriate strategy for each case. Because simultaneous dual injection is absent, good-quality angiography is even more important than in the classic algorithm; when in doubt, a “stepwise double injection” (sequential injection of the donor artery and then the CTO artery through a single access site) is recommended before PCI. The algorithm comprises five variants (A–E), chosen according to the intended approach, the feasibility of retrograde crossing, and the presence of interventional collaterals [8]. The MHA algorithm is shown in Figure 1. Overall, MHA aims to use smaller sheaths, avoid transfemoral access when possible, and reduce the need for dual access.

All five variants start with a single 6 or 7 Fr sheath placed via forearm access (radial, ulnar, or distal radial artery). Unlike in the classic hybrid algorithm, simultaneous dual-contrast injection is not mandatory. The variant chosen depends on the intended crossing technique and on the type of collaterals available to the operator.

In variant A, the procedure begins with an antegrade wire escalation attempt through a single 6 Fr sheath, typically performed as “blind” wiring with a soft, low-penetration-force hydrophilic guidewire and without full visualization of the distal segment. Contralateral collaterals remain available should this attempt fail. If antegrade crossing fails, the operator switches to the donor vessel to seek a retrograde route through the same access site. Successful collateral crossing is followed by placement of a second 6 Fr sheath contralaterally to complete the procedure by retrograde wire escalation (RWE) or RDR. Failure to cross the collaterals calls for a second 7 Fr sheath to escalate antegrade techniques with full vessel visualization. Variant B follows the same initial steps but applies when no suitable contralateral collaterals exist. Failure of the “blind” antegrade attempt leads directly to escalation of antegrade techniques (a stiffer guidewire or ADR), again with a second 7 Fr sheath placed contralaterally. The original 6 Fr access is then used to place a guiding catheter in the donor vessel, which allows contralateral (dual) injection.

When contralateral collaterals represent the main route for interventional access, variant C is used. A single 6 Fr sheath is first used to attempt collateral crossing. Success is followed by placement of a second 6 Fr sheath contralaterally to pursue a retrograde strategy; failure requires escalation to antegrade techniques through a second 7 Fr sheath. Variant D applies when no interventional collaterals are available and ADR is the intended technique. Because ADR requires simultaneous dual injection for adequate visualization of the vessel course, dual access (7 and 6 Fr sheaths) is established from the outset, before the first crossing attempt. All remaining techniques stay available should ADR fail. Variant E is used when ipsilateral collaterals are the main source of visualization. A single 7 Fr sheath is generally sufficient and allows all four crossing techniques. Only failure with a single catheter prompts placement of a second 6 Fr sheath contralaterally to introduce a second catheter into the same vessel for a “ping-pong” technique [8].

However, the MHA algorithm promotes blind wiring, which departs from one of the main safety principles of CTO PCI: simultaneous contralateral dual injection. The lack of visualization can increase procedural uncertainty and the risk of perforation. To limit this risk, blind wiring is restricted to soft, low-penetration-force guidewires, and intermediate- or high-penetration-force wires should be avoided in this setting. For this reason, the algorithm is recommended only for very experienced CTO operators working in high-volume centers. A further weakness is that obtaining a second access site during the intervention, after full-dose heparin has been administered, may increase the risk of access-site bleeding.

The efficacy and safety of the MHA algorithm have been evaluated in one retrospective and one prospective single-center study. Agostoni et al. showed that the algorithm is feasible and can be highly successful when used appropriately, but that success depends on patient and lesion selection, operator expertise, and institutional experience. MHA was prospectively attempted in 56 consecutive CTO PCI procedures in 54 patients, with a mean J-CTO score of 2.04 ± 0.95. Lesion-based technical success was 94.6% (53/56; three failures, two of them in the same patient), and procedural success was 91.1% (51/56; the two additional failures were one tamponade requiring pericardiocentesis and one periprocedural myocardial infarction). Patient-based technical and procedural success was 98.1% (53/54) and 94.4% (51/54), respectively. In 34 procedures (60.7%), a single-catheter approach was used (always trans-wrist, except for one single-femoral case). Of the 22 procedures (39.3%) using dual catheters, one was bifemoral and the rest were bi-wrist. Antegrade wiring was the successful technique in 29 cases (51.8%), ADR in 5 (8.9%), and the retrograde approach in 19 (33.9%) [9]. In the retrospectively analyzed single-center registry of Zivelonghi et al., 91 of 100 consecutive CTO PCI patients treated between March 2016 and October 2017 were approached according to the algorithm (mean J-CTO score 1.9 ± 1.2; J-CTO score ≥ 3 in 34%). Procedural success was 89%; 39 patients were treated with dual catheters, and the remaining 52 with a single catheter. A perforation rate of 2.2% and no vascular complications were reported [10].

Overall, MHA may be useful in some less complex CTO cases, especially those with ipsilateral collaterals that are handled by experienced operators. However, dual access remains the gold standard for challenging CTOs, such as those requiring a retrograde approach or ADR.

## 4. The Cut Balloon Technique (CBT)

Coronary artery perforation remains one of the most serious complications of CTO PCI. In the OPEN-CTO registry, which included 1000 patients undergoing percutaneous revascularization of a CTO, clinically relevant perforation was the most frequently reported major in-hospital complication occurring in 4.8% of procedures, compared with an overall rate of major adverse cardiac and cerebrovascular events (MACCEs) of 7% [11]. Given the potentially life-threatening nature of this complication, an immediate and effective response by the operator is essential.

Before techniques for managing a perforation once it has occurred are discussed, it should be emphasized that prevention remains the most effective strategy. A thorough pre-procedural risk–benefit assessment, accounting for lesion complexity and operator experience, should always precede the choice of crossing strategy, particularly for higher-risk techniques such as retrograde or ADR approaches. Careful selection of a technique appropriate to the risk of the individual case, rather than proficiency in managing complications after the fact, remains the foundation of safe CTO PCI practice. When patient selection and procedural conduct are appropriately careful, the need for techniques such as CBT should arise infrequently.

**Figure 2:**
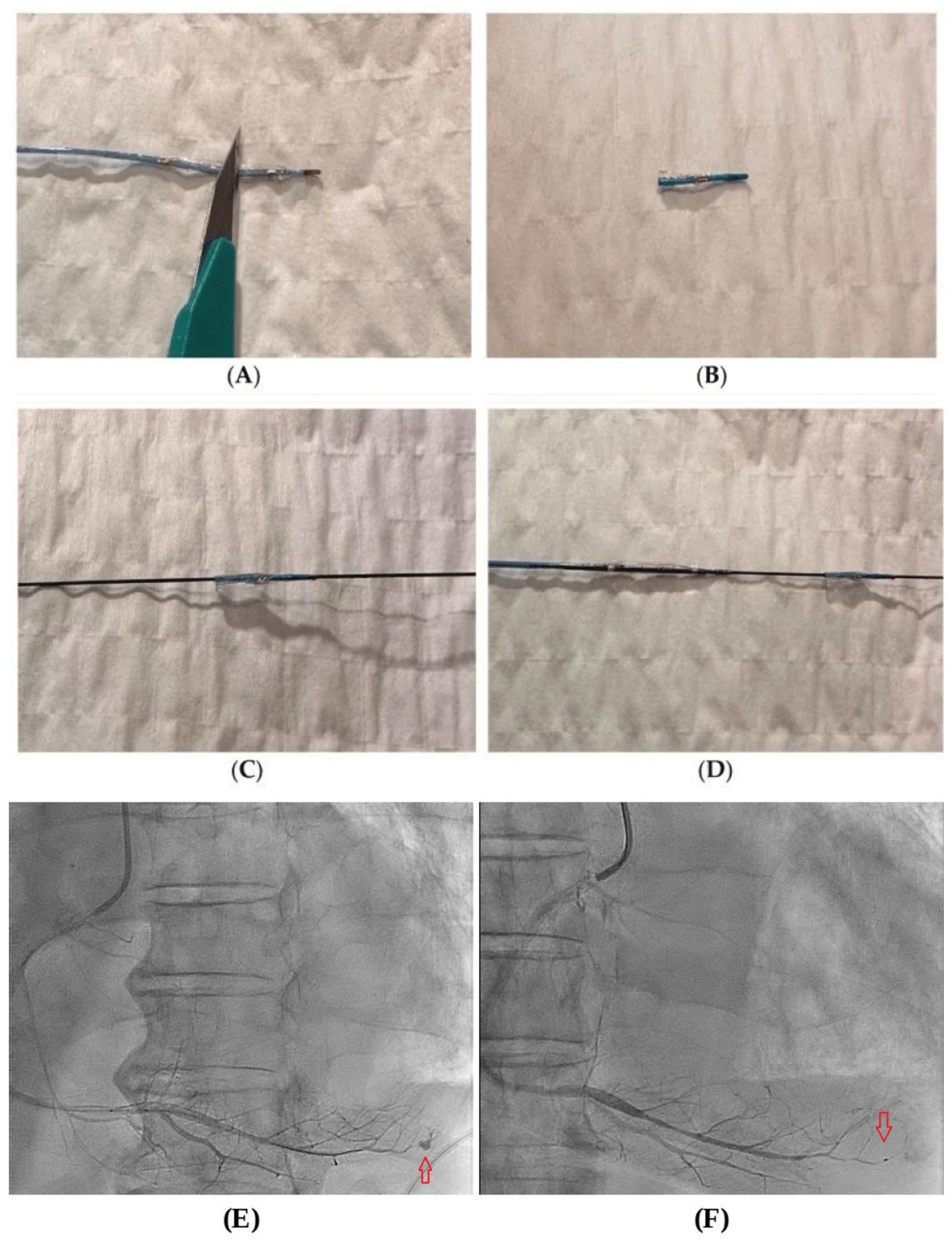
The cut balloon technique (CBT) for sealing a distal coronary perforation. (**A**) A previously inflated angioplasty balloon is cut in the middle with surgical scissors. (**B**) The distal balloon remnant, still carrying its radiopaque marker, is separated from the rest of the shaft. (**C**,**D**) The balloon remnant is threaded back onto the guidewire alongside a second angioplasty balloon, allowing the two to be advanced together as a single assembly toward the perforation site. (**E**) Intraprocedural fluoroscopic image showing contrast extravasation at the site of distal coronary perforation (red arrow). (**F**) After advancement and deployment of the cut balloon remnant, fluoroscopy confirms sealing of the perforation, with no further contrast extravasation (red arrow). Source: own collection, Cardiology Department, 1st Military Hospital in Lublin.

Iatrogenic distal coronary perforation is a potentially life-threatening complication that occurs more often during CTO PCI. The use of coils, thrombin, clot, fat, or tissue glue is either difficult or expensive because it requires microcatheters for target deployment [12,13]. CBT, by contrast, is reported to be simple and inexpensive, and does not appear to require extensive operator experience. It was first described in 2020 in a single-center series of six patients [14]. The technique is illustrated in Figure 2. The technique involves cutting a previously inflated balloon in the middle, taking the distal part of the balloon, and mounting it on the angioplasty wire alongside another balloon. The two are then advanced together to the site of distal perforation. Thanks to the radiopaque marker on the cut remnant of the balloon, it can be navigated easily under fluoroscopy. Sometimes several balloon remnants are needed to seal the perforation. A disadvantage of the technique is the risk that, in calcified or tortuous vessels, the balloon may become stuck along the way; in this case, it can be retrieved using a guide extension catheter.

In a recent multicenter study, the authors included 26 patients with distal vessel perforations treated with CBT, 3 of whom were treated during CTO PCI. The balloons used for vessel closure were mostly those previously used for lesion preparation, with diameters ranging from 1.5 to 4.0 mm. Most cases require one or two balloons to close the perforation. The in-hospital prognosis was favorable, with cardiac tamponade requiring pericardiocentesis in only four patients (15.4%). No emergency surgery or cardiac death was observed [15]. CBT therefore appears to be a feasible, low-cost option for sealing distal perforations, but the evidence is limited to small series, and the rate of tamponade requiring pericardiocentesis (4/26 patients) shows that it does not replace prompt recognition of perforation and a prepared bailout strategy.

## 5. The Condom Technique

**Figure 3:**
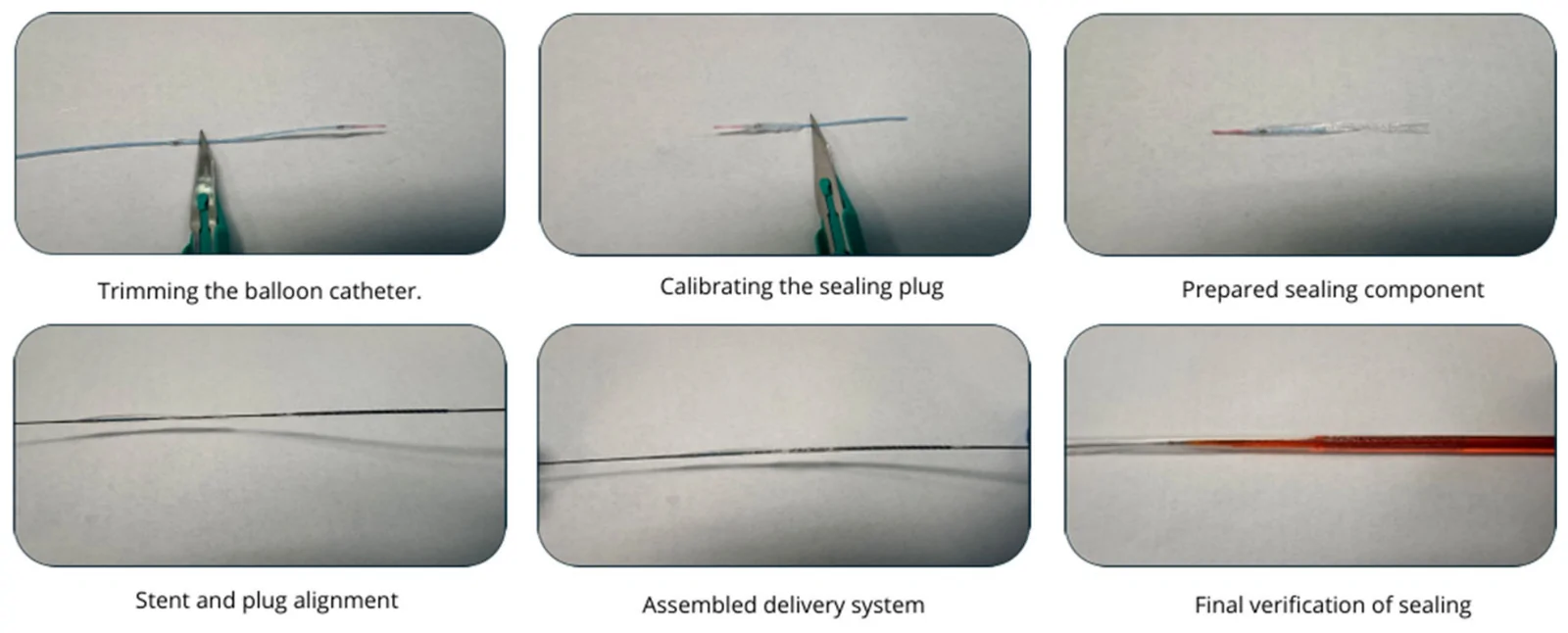
Stepwise representation of the condom technique: trimming the balloon catheter, calibrating the sealing plug, preparing the sealing component, aligning the stent and the plug, assembling the delivery system, and final verification of sealing. Source: own collection, Cardiology Department, 1st Military Hospital in Lublin.

The need for inexpensive, rapidly deployable vascular-sealing methods remains very important in the management of distal coronary perforations. As a modification of CBT, Agostoni et al. recently presented the so-called “condom technique”. This approach involves mounting a stent on the cut distal remnant of a balloon, advancing the assembly over the guidewire to the distal vessel segment, and then implanting the stent to achieve vessel closure [16]. The stepwise preparation of the system is shown in Figure 3. The evidence for this technique consists of a technical report without outcome data, and the implications of leaving a permanent stent in the distal vessel for antiplatelet therapy and long-term patency have not been studied.

## 6. Tip-Detection Antegrade Dissection and Re-Entry (TD-ADR)

Two broad categories of ADR exist: device-based and wire-based. Wire-based ADR includes techniques such as subintimal tracking and re-entry (STAR), mini-STAR, and limited antegrade subintimal tracking (LAST). Device-based ADR uses dedicated systems, such as dual-lumen catheters (e.g., ReCross) or the Stingray balloon, to facilitate re-entry into the true lumen of the vessel. Classically, Stingray-ADR relies on angiographic guidance, but its efficacy may be limited in selected anatomies. IVUS imaging can address this limitation by confirming the true-lumen position of the wire after the cap has been punctured and, when contralateral injection is not feasible, by confirming the location of the guidewire tip.

**Figure 4:**
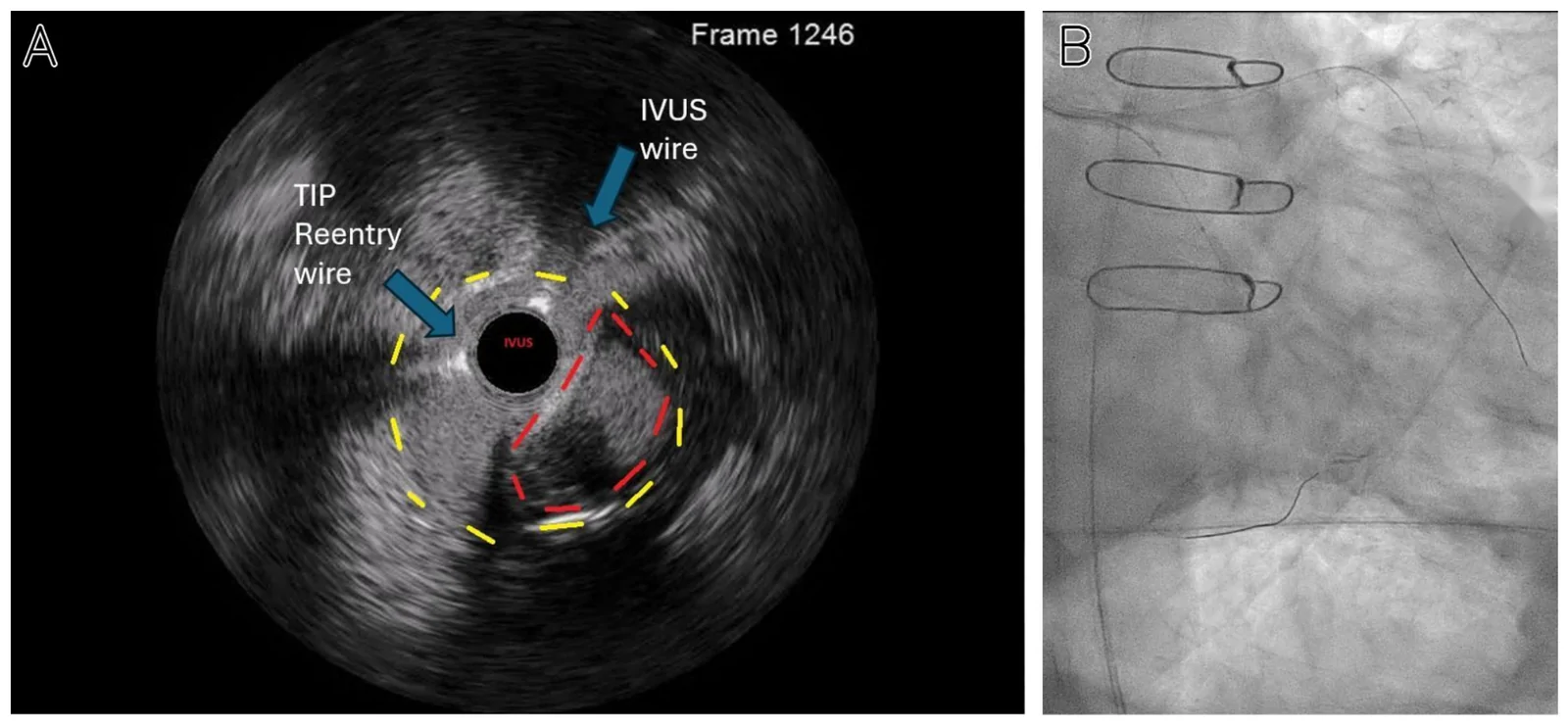
Tip-detection antegrade dissection and re-entry (TD-ADR) under intravascular ultrasound (IVUS) guidance. (**A**) IVUS cross-sectional image showing the IVUS wire and the TIP re-entry wire within the vessel. The true lumen is outlined in red, and the subintimal space (false lumen) is outlined in yellow. (**B**) Corresponding fluoroscopic image showing the wire and balloon marker positions along the vessel course. Source: own collection, Cardiology Department, 1st Military Hospital in Lublin.

TD-ADR combines IVUS and angiography to direct a re-entry wire into the true lumen without a dedicated re-entry system. Figure 4 illustrates IVUS-guided re-entry. The first human case report of TD-ADR was published in 2022 [17]. A subsequent case report described TD-ADR with integrated fluoroscopic and IVUS images, merging both modalities to direct the guidewire to the true lumen quickly and precisely [18]. The method can be challenging and time-consuming even for experienced operators, and the second operator manipulating the IVUS probe is as important as the first. More recently, a retrospective four-center comparison of 27 TD-ADR cases performed with a dedicated high-penetration re-entry wire and 27 Stingray-ADR cases reported a higher ADR success rate with TD-ADR (100% vs. 67%) and a shorter median procedure time (145 vs. 185 min), with few in-hospital major adverse events in either group [19]. Nevertheless, the evidence remains limited to small retrospective series and case reports, and prospective studies are needed to establish its applicability.

## 7. HydroDynamic Contrast Recanalization (HDR), or the Modified Carlino Technique

Injection of contrast within a CTO lesion has been an option for treating an uncrossable proximal cap by hydraulic dissection since 2008, when the contrast-guided STAR technique was published. That technique was based on the forceful injection of large amounts (3–4 mL) of contrast to recanalize the occlusion hydraulically, which resulted from tears in the intima separating the false and true lumens. However, it was accompanied by extensive dissections and unpredictable true-lumen re-entry, and restenosis rates at follow-up tended to be high. For these reasons, the technique was abandoned [20].

The idea was not completely abandoned, however. The technique was improved and is now known as the “Carlino technique”. It involves injecting a small amount of contrast through a microcatheter for two main reasons. First, when the microcatheter is intraplaque, contrast follows the path of least resistance and modifies plaque compliance by softening it, which facilitates wire crossing. Second, if the microcatheter is extraplaque, two angiographic patterns may be observed. A “storm cloud” appearance usually indicates contrast injection into a small branch that becomes transiently visible; the wire should not be advanced through such a branch because of the high risk of perforation. In contrast, “tubular staining” indicates contrast accumulation in a larger vessel along subintimal planes, where the wire can be advanced or redirected safely to cross the lesion. To date, the Carlino technique has been increasingly used by experienced operators for uncrossable, calcified lesions [21]. In a recent multicenter registry of 4508 CTO PCI cases that used ADR or the retrograde approach, the Carlino technique was applied in 128 (2.8%), predominantly in more complex lesions. Its use was associated with lower technical success (65.6% vs. 78.5%) and a higher perforation rate (18.0% vs. 8.9%) than in cases without it, although procedural success was higher when it was used for retrograde crossing (81.5% vs. 58.4%) [22].

**Figure 5:**
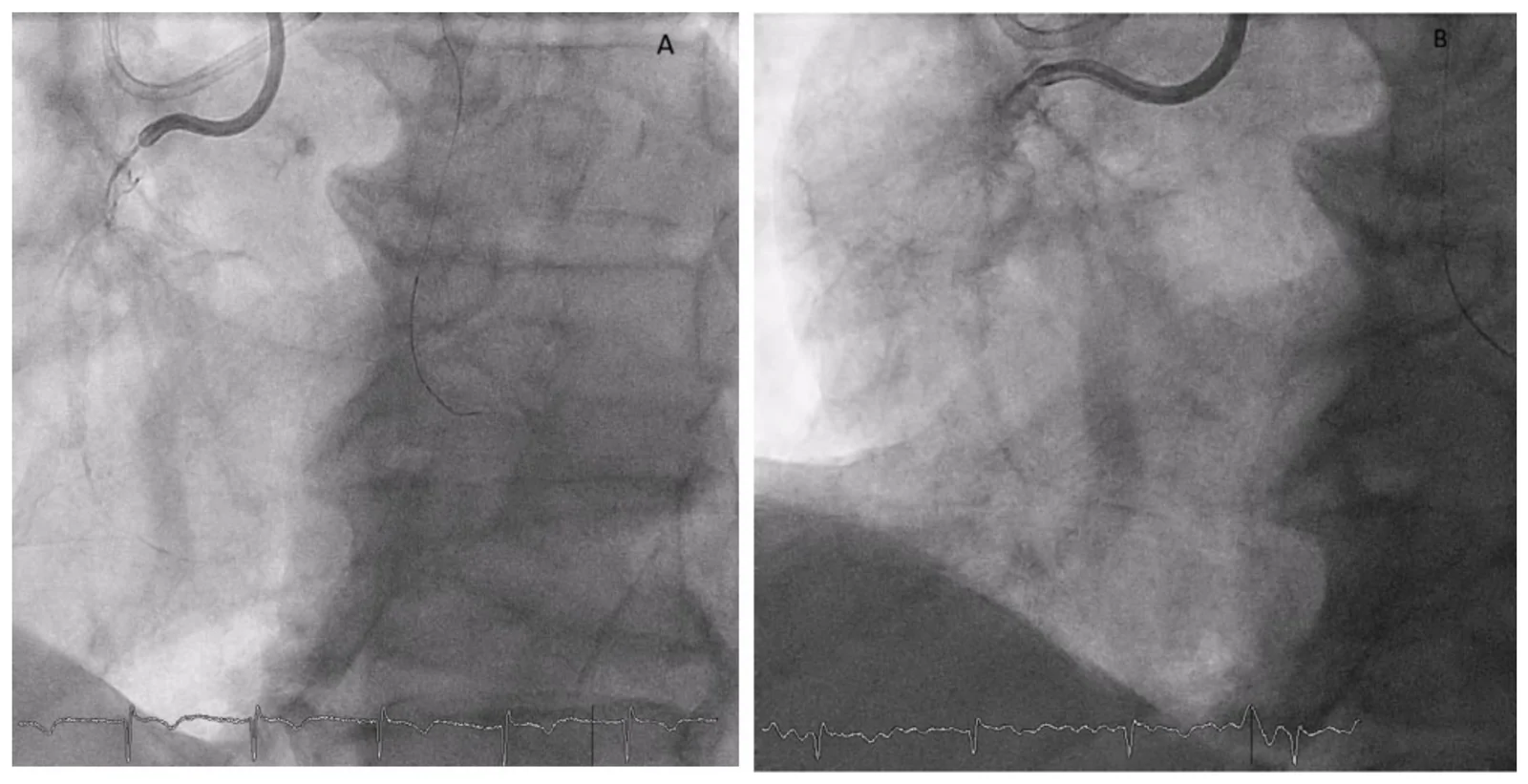
HydroDynamic contrast recanalization (HDR) technique. (**A**) Puncture of the proximal CTO cap with a polymer-jacketed guidewire, confirmed in two orthogonal projections. (**B**) Advancement of the microcatheter intraplaque within the CTO body and administration of a small contrast volume (0.5 mL) to visualize the direction for further guidewire penetration. Source: own collection, Cardiology Department, 1st Military Hospital in Lublin.

Carlino et al. later modified the technique and renamed it HydroDynamic contrast recanalization (HDR). HDR is based on the synergistic use of contrast microinjections and polymer-jacketed wires. The main difference is a shift in procedural philosophy: in HDR, contrast—not the wire—leads the way. The technique is illustrated in Figure 5 and consists of the following steps:

1. Puncture of the proximal CTO cap with a polymer-jacketed CTO wire (confirmed in orthogonal projections).
2. Avoidance of distal progression of the guidewire.
3. Advancement of the microcatheter intraplaque into the CTO body.
4. Gentle, slow administration of a small amount of contrast (0.5 mL).
5. The contrast visualizes the CTO, indicates its direction, and facilitates penetration of the guidewire.

This technique may be particularly effective for long occlusions, calcified lesions, ambiguous proximal caps, diseased distal vessels, and poor collateral circulation, in which antegrade wiring, retrograde strategies, and re-entry techniques can be challenging. Carlino et al. included 43 patients with 45 CTOs treated with HDR. The mean J-CTO score was 2.3, and the mean PROGRESS-CTO score was 1.8. Ambiguous or blunt caps were present in 34 lesions, lesions longer than 20 mm in 27, and moderate or heavy calcification in 36. Procedural success with HDR was 100%, with no complications reported in this single-center series [23]. According to its authors, HDR is reproducible and can be repeated during a single procedure, but independent confirmation is lacking. Of note, the broader registry of the Carlino technique showed lower technical success and more perforations, without adjustment for lesion complexity, so the safety of contrast-guided crossing must be established prospectively. However, given the small sample size and the potential for selection bias, these results should be interpreted with caution, and larger studies, ideally randomized, are needed to define the role of HDR in routine clinical practice.

## 8. The Microcatheter Anchor Technique (MAT)

**Figure 6:**
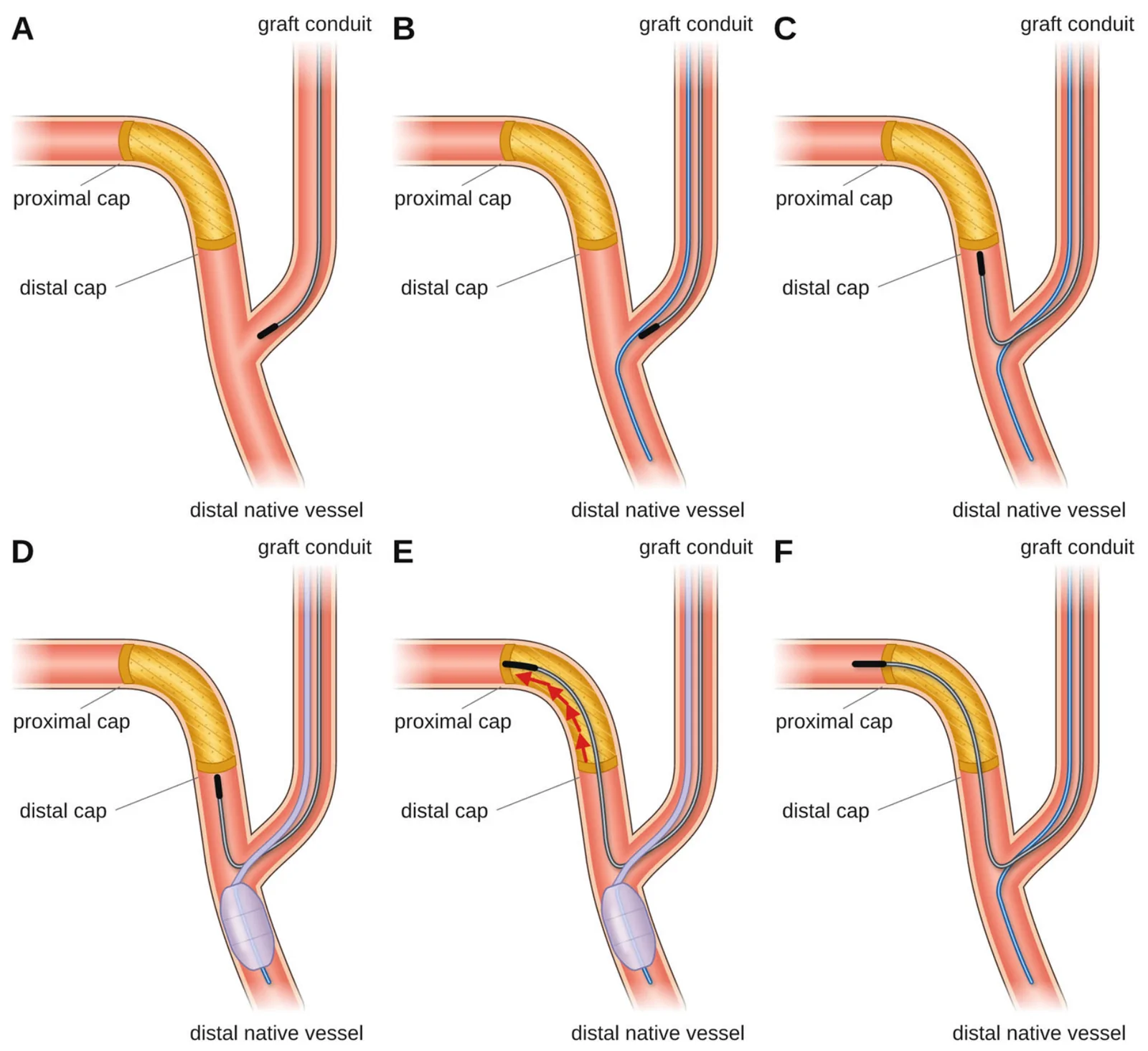
Stepwise representation of the microcatheter anchor technique (MAT) in post-CABG anatomy with a retroflexed graft insertion. (**A**) Baseline anatomy: the microcatheter approaches the distal cap at the retroflexed graft conduit anastomosis. (**B**,**C**) The microcatheter and support wire are advanced further into the distal native vessel. (**D**) An anchor balloon is inflated within the distal native vessel to stabilize the microcatheter and prevent prolapse. (**E**) With the balloon inflated, stabilizing force (red arrows) is transmitted to the microcatheter, improving pushability toward the distal cap. (**F**) The stabilized assembly successfully penetrates the distal cap, enabling guidewire crossing [25].

CTO PCI in post-CABG patients is challenging because acute angles at the graft anastomosis often prevent retrograde passage of the microcatheter through the retroflexed vessel and may lead to microcatheter prolapse [24]. Iqbal et al. described the microcatheter anchor technique (MAT) to address this problem. The procedural sequence is illustrated in Figure 6. The key initial step is successful delivery of the microcatheter tip into the retroflexed vessel. Once the tip is positioned, an anchor balloon sized 1:1 to the vessel diameter is inflated to prevent microcatheter prolapse and to provide support for wire penetration of the distal cap. The reported advantages of MAT include stabilization of the microcatheter at acutely angulated anastomoses, facilitation of hard distal cap penetration, feasibility in a 6 Fr system, and potential use outside CTO PCI in severely angulated vessels. Potential limitations include ischemia during prolonged balloon inflation and perforation if the balloon is incorrectly sized. Overall, MAT is a promising approach to improve microcatheter support at retroflexed graft insertion sites; however, the available evidence is limited to a single case report, and its effectiveness and safety remain to be confirmed [25].

## 9. The RODIN-CUT Technique

**Figure 7:**
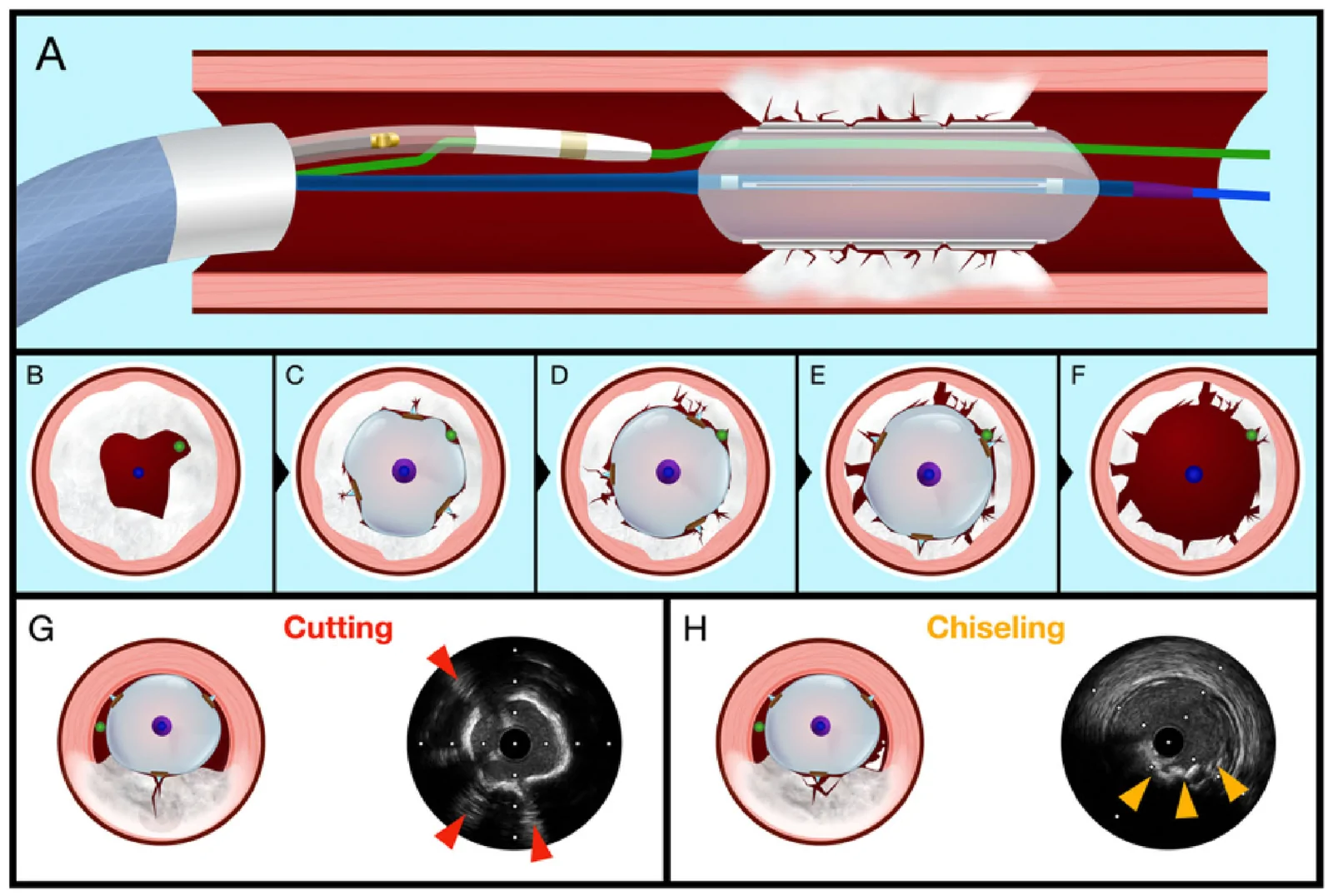
(**A**) The IVUS catheter positioned proximal to the cutting balloon enables rapid switching between imaging and therapy. (**B**–**F**) Sequential cutting balloon inflations progressively modify the lesion; blue and green dots mark the two guidewires. (**G**) “Cutting” effect with plaque fragmentation. (**H**) “Chiseling” effect showing superficial fissures after repeated inflations [26].

The RODIN-CUT technique is a systematic protocol of sequential cutting balloon (CB) inflations under real-time IVUS guidance, designed to fracture severely calcified plaques. The technique is illustrated in Figure 7, and its steps are as follows:

1. Access: a 7 Fr guiding catheter is used, and the target vessel is double-wired.
2. Baseline assessment: an initial IVUS pullback is performed to document lesion morphology.
3. High-pressure inflation: the CB is inflated at 16–18 atm (up to 20 atm) for 5–10 s.
4. Real-time imaging: the balloon is advanced, and plaque fractures are assessed by IVUS immediately after each inflation.
5. Sequential cycle: the process is repeated at least five times (up to 15), using blade rotation and cumulative mechanical stress to achieve a “chiseling” effect and complete plaque fragmentation.

CB sizing is crucial: for safety, the balloon should be 0.5 mm smaller than the media-to-media diameter. The procedural goal is a residual stenosis of <30% with Thrombolysis in Myocardial Infarction (TIMI) 3 flow in the distal vessel. Ungureanu et al. described two main effects of the technique, “cutting” and “chiseling”, both of which can be identified by IVUS [26].

The main disadvantage of the technique may be the limited deliverability of the CB, which restricts its use to larger vessels. Moreover, the shadow cast by the second guidewire may make it harder to identify and count the fissures. Although standard recommendations advise CB inflation at nominal pressure (6 atm), higher-pressure inflations have been shown to improve plaque modification in calcified lesions. Evidence from the COPS randomized trial demonstrated that high-pressure CB use is safe and yields a larger minimal stent area (MSA) and reduced stent eccentricity compared with conventional balloon predilation [27]. The single-arm PREPARE-CALC-COMBO study reported greater in-stent acute lumen gain and a larger MSA after rotational atherectomy followed by cutting balloon angioplasty than in a previous cohort treated with rotational atherectomy or modified balloons alone [28]. However, the randomized ROTA-CUT trial (60 patients) found a similar MSA after rotational atherectomy followed by a cutting balloon versus a non-compliant balloon [29]; possible explanations, such as inflation pressure and lesion length, remain speculative. Importantly, high-pressure inflations did not increase major complications, probably because CBs create controlled incisions that reduce the risk of uncontrolled dissections. Proper balloon sizing remains essential, as the goal is to fracture calcified plaque while minimizing stress on the surrounding vessel wall [28]. Nevertheless, the evidence supporting this technique remains preliminary.

## 10. Ostial RODIN-CUT Angioplasty and Stenting (ORCAS)

**Figure 8:**
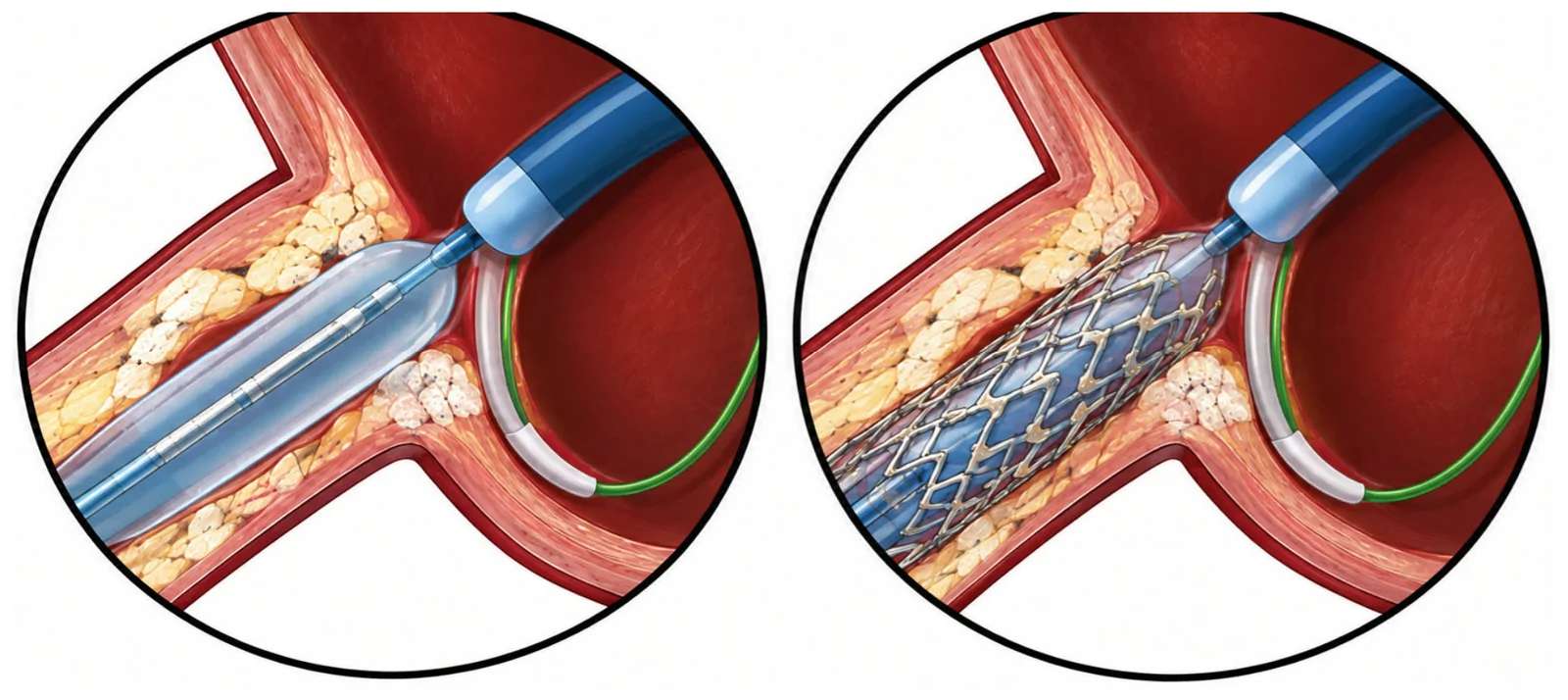
The left panel illustrates cutting balloon inflation using the RODIN-CUT technique, which effectively modifies a calcified ostial lesion. The right panel shows real-time IVUS-guided stent placement, with the IVUS catheter positioned at the right coronary artery ostium to ensure precise alignment and prevent geographic miss. Source: own collection, Cardiology Department, 1st Military Hospital in Lublin.

ORCAS (Ostial RODIN-CUT Angioplasty and Stenting) is an advanced procedural approach designed for the treatment of complex, heavily calcified aorto-ostial coronary lesions. It should be noted that it is not a distinct CTO-crossing strategy: the guidewire is advanced using a conventional antegrade technique, and the method addresses only the steps that follow vessel access. ORCAS derives from the RODIN-CUT method, using sequential plaque preparation with real-time IVUS guidance to ensure precise stent deployment. Figure 8 illustrates lesion preparation and stent positioning.

The lesion is modified using sequential, high-pressure (e.g., 18 atm) cutting balloon inflations. After each inflation, real-time IVUS pullbacks are performed to assess the extent of plaque fragmentation and calcium fracture. This iterative process continues until optimal lesion modification—evidenced by a significant increase in the minimal lumen area (MLA)—is achieved. To avoid geographic miss, a second guidewire carries the IVUS transducer into the aortic cusp, aligning it directly in front of the coronary ostium. This provides a continuous, direct view of the anatomical landmarks during stent positioning. The operator then adjusts the stent position under live IVUS visualization to ensure full ostial coverage while strictly minimizing aortic protrusion (ideally <1 mm).

Continuous imaging thus reduces reliance on angiography alone, which is often unreliable because of complex ostial takeoffs. The technique is intended to facilitate a larger MSA through meticulous plaque preparation and confirmed balloon expansion, although this has not been demonstrated in comparative studies. By providing real-time feedback, ORCAS may help prevent stent malapposition and other complications associated with suboptimal lesion preparation in calcified anatomy. Implementation of ORCAS requires a large-bore guide catheter, such as 7 Fr, to accommodate the IVUS probe and the therapeutic devices, including the cutting balloon or stent, simultaneously [30]. Ostial stent positioning can also be managed using the Wire in Aorta for Localization and Protection of the Ostium (WALPO) technique described by Leibundgut et al. [31]. The main differences between ORCAS and WALPO are summarized in Table 1; this comparison reflects the authors’ synthesis of case-level descriptions, as no head-to-head data exists.

## 11. Plasmawire

Plasmawire™ (RetroVascular Inc., Pleasanton, CA, USA) is a novel bipolar radiofrequency (RF) wire system that uses plasma-mediated ablation to facilitate CTO crossing. The system uses two independent wires to create a channel by delivering RF energy between their tips. Kanno et al. reported the use of Plasmawire in seven CTO PCI cases. All procedures were successful, with a single RF application required in most cases (5/7). No complications occurred during hospitalization or at 30-day follow-up. The authors noted that device efficacy depends on the distance between the wires, with the best results achieved when the gap is less than 2 mm, and on tissue characteristics, as heavily calcified tissue was associated with poorer outcomes [32]. Plasmawire was reported to be effective in both retrograde and antegrade re-entry in this small first-in-human series, but a 3D angiographic map is essential during the procedure. Despite these promising early results, the data come from a small first-in-human series, and larger prospective clinical studies are needed to confirm the applicability of the Plasmawire™ system in everyday practice.

## 12. CTCA in CTO PCI

CT coronary angiography (CTCA), a non-invasive imaging method, plays an important role in the diagnosis and management of coronary artery disease, including CTOs. The introduction of multi-slice CT and 3D volume rendering has enhanced the ability of CTCA to provide high-quality images and a detailed assessment of plaque characteristics [33]. Beyond detecting stenosis, CTCA can assess plaque composition and three-dimensional vessel structure, supporting pre-procedural planning for PCI. It helps identify features that affect PCI success, such as calcification, tortuosity, and lesion length, and can predict outcomes using individual or combined scoring systems [34]. The CT-RECTOR score is a non-invasive prediction tool developed to estimate the likelihood of successful guidewire crossing of a CTO within 30 min during PCI. Derived from a multicenter registry of 240 CTO lesions, it uses CT-derived anatomical and clinical features to provide an objective measure that minimizes reliance on operator skill [35]. In the original CT-RECTOR cohort, the score predicted successful guidewire crossing within 30 min better than the angiographic J-CTO score, which remains the reference standard (area under the curve 0.83 vs. 0.71) [35].

Comparing Table 2 and Table 3, which come from different cohorts (the head-to-head comparison is the one in the CT-RECTOR cohort described above), suggests a similar trend: the higher the score, the lower the likelihood of successful guidewire crossing within 30 min. For CT-RECTOR, the success rate decreases from 95% in the “easy” group to 22% in the “very difficult” group. For J-CTO, a comparable decline is seen, from approximately 88–92% to 10–22%, depending on whether the derivation or the validation dataset is considered. Both scores rely on similar anatomical lesion features, such as proximal cap morphology, occlusion length, and vessel tortuosity. CT-RECTOR stands out, however, in that all these features can be assessed non-invasively, even before diagnostic coronary angiography is performed. This makes it particularly useful at the stage of patient selection and procedural planning, before the operator reaches the catheterization laboratory. J-CTO, by contrast, remains the angiography-based gold standard, assessed immediately before or during the procedure itself, although it can also be estimated from CTCA, as discussed below [36].

Despite growing evidence supporting the usefulness of CTCA in CTO PCI planning, the method is, to our knowledge, not yet formally recommended as a standard element of procedural preparation in the major myocardial revascularization guidelines. The topic is, however, widely discussed in expert literature. A recent systematic review and meta-analysis (47 studies included in the systematic review) reported a high degree of agreement between CTCA and invasive angiography when the J-CTO score was applied and concluded that CTCA is a safe and effective pre-procedural tool [36]. A similar position is taken in the consensus statement of the EAPCI, the EACVI, and the ESC Working Group on Cardiovascular Surgery on the evaluation of patients with CTO considered for revascularization [38]. In clinical practice, CTCA is increasingly used in high-volume referral centers to plan the crossing strategy, although its use currently remains a matter of expert best practice rather than a formal guideline requirement.

## 13. New Calcium-Modifying Technologies

Calcified lesions remain a major challenge during CTO procedures. Although several calcium-modifying strategies are available, including intravascular lithotripsy (IVL), super-high-pressure balloons (SHPs; best known in clinical practice under the trade name OPN), orbital atherectomy (OA), and rotational atherectomy (RA), each has important limitations. This unmet need has stimulated the development of new approaches for increasingly complex CTO cases.

### 13.1. Shockwave Javelin^®^

**Figure 9:**
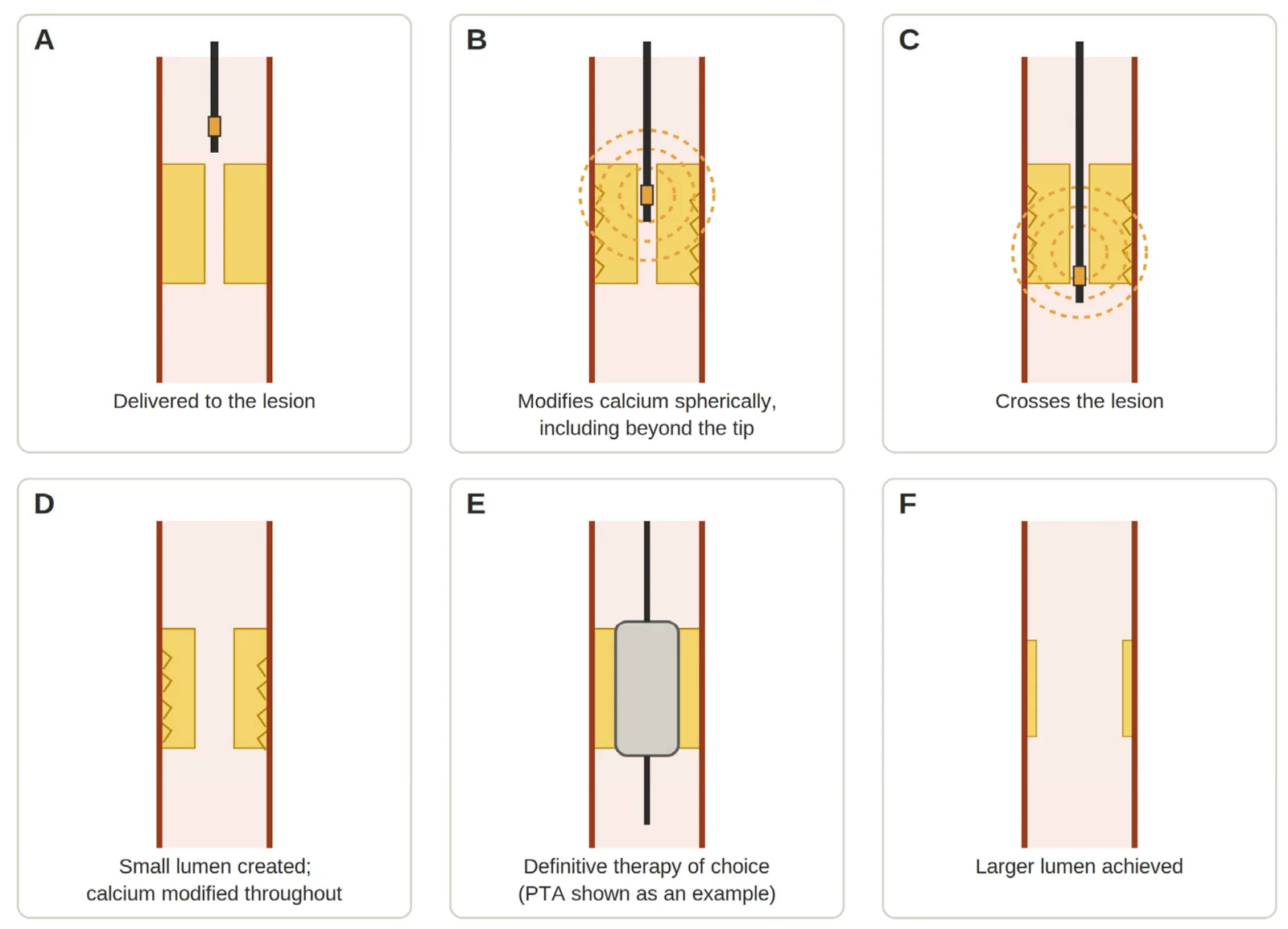
Step-by-step mechanism of the Shockwave Javelin™ system. (**A**) The device is delivered to the lesion. (**B**) It modifies calcium spherically, including beyond the catheter tip. (**C**) Javelin crosses the lesion. (**D**) A small lumen is created, with calcium modified throughout the treated segment. (**E**) Calcium modification facilitates definitive therapy of choice, with percutaneous transluminal angioplasty (PTA) shown as an example. (**F**) A larger lumen is achieved.

The Shockwave Javelin (Shockwave Medical, Inc., Santa Clara, CA, USA) is a forward-firing intravascular lithotripsy (IVL) catheter designed to treat severe calcification and to cross difficult lesions. Its mechanism is illustrated in Figure 9. Unlike traditional IVL devices that rely on balloon inflation, it carries a single sonic pulse emitter near the distal tip and uses a fluid-filled window to transmit energy directly into the lesion as the device is gently advanced over a 0.014-inch guidewire so that calcium modification and crossing can be combined. The peripheral version is already in clinical use and, according to the manufacturer, can deliver up to 120 pulses [39]. A coronary version was evaluated in a prospective, multicenter (three UK centers), single-arm, first-in-human, early feasibility study, reported as a research letter, in 20 patients with de novo, moderately to severely calcified lesions with ≥90% stenosis, most of whom had a CTO (11 of 20). Procedural success was 90%, and the primary safety endpoint was met in 90% of patients (the only adverse events were two periprocedural non-Q-wave myocardial infarctions). Javelin alone was sufficient for crossing and calcium modification in 65% of patients, an additional balloon-based IVL device was needed in the remaining 35%, and one patient required bailout atherectomy [40]. This data come from a very small, non-randomized series and cannot define efficacy or safety, particularly in CTO. The pivotal single-arm FORWARD CAD study (up to 158 patients) was initiated in 2025 [40]; its results and CTO-specific data are awaited.

### 13.2. Hertz Contact IVL (LithiX)

**Figure 10:**
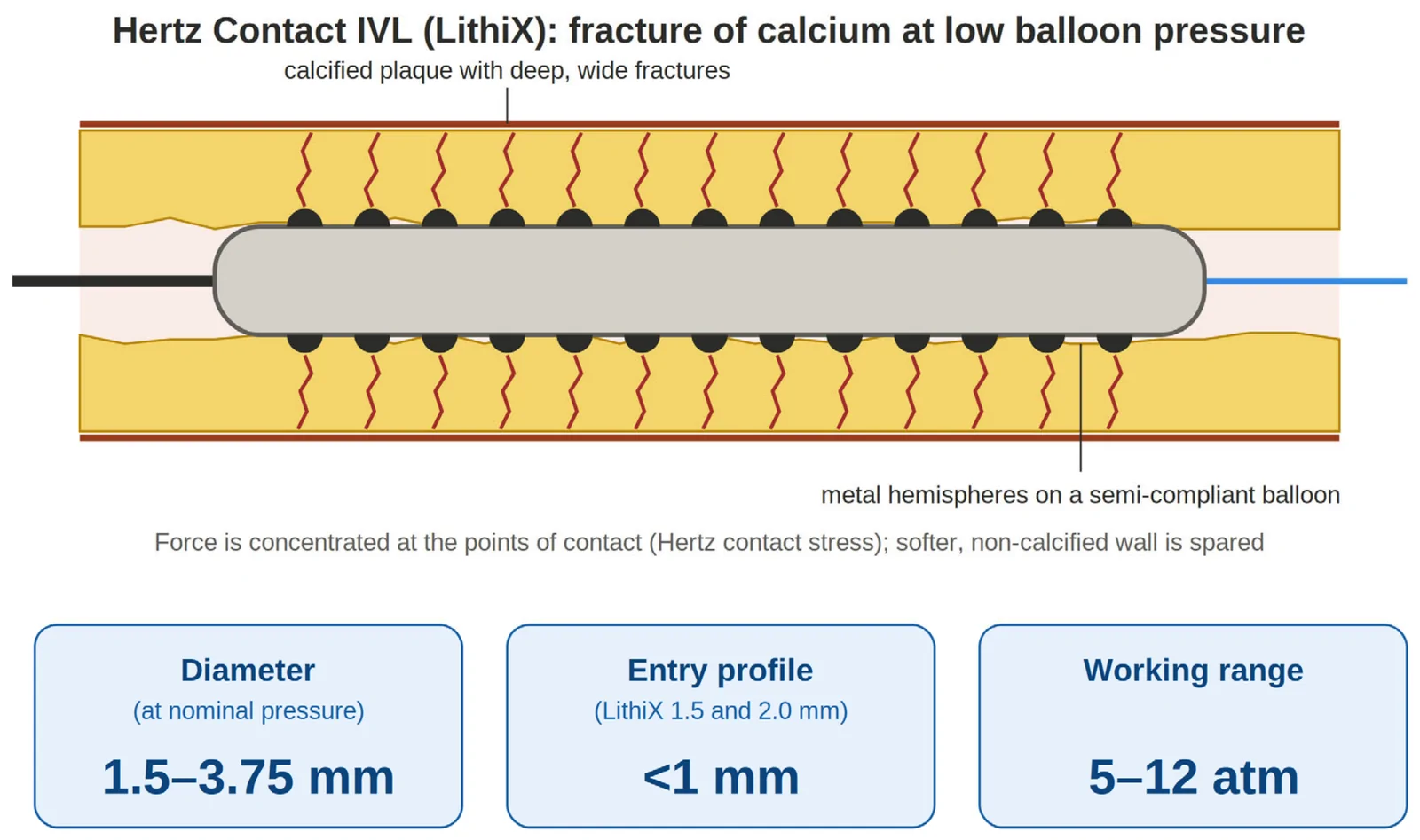
Design and working parameters of the Hertz Contact IVL (HC IVL) system (LithiX). The catheter incorporates low-profile metal hemispheres on a semi-compliant balloon and is reported to be effective at low working pressures: diameter range 1.5–3.75 mm at nominal pressure, entry profile <1 mm for the 1.5- and 2.0-mm LithiX catheters, and working range 5–12 atm.

The LithiX Hertz Contact IVL System (Elixir Medical Corporation, Milpitas, CA, USA) is a transcatheter device that uses low-profile metal hemispheres on a semi-compliant balloon to treat calcified arteries. Figure 10 shows the device design and working parameters. When inflated at low pressure, these hemispheres concentrate force at the points of contact with calcium and use Hertz contact stress mechanics to create deep, wide fractures in calcified tissue while minimizing damage to softer, non-calcified vessel walls, without an external energy source. In the multicenter, prospective, single-arm PINNACLE-I trial (60 patients, 63 moderately to severely calcified de novo lesions), the primary endpoint (residual stenosis < 50% without in-hospital MACE) was achieved in 98.3%, and residual stenosis was <30% in all lesions after stenting. The 30-day MACE rate was 1.7% (one periprocedural non-Q-wave myocardial infarction); no cardiovascular deaths, definite or probable stent thromboses, or device-related events occurred up to 6 months [41]. In the OCT substudy (32 patients), calcium fractures were detected in 90.6% of lesions (mean fracture depth 0.81 ± 0.33 mm), and stent expansion at the minimal stent area site was 96.7 ± 25.5% [41]. LithiX received CE mark in 2025 [42]. Taken together, these findings suggest effective calcium fracture and a favorable short-term safety profile. However, the data comes from a single industry-sponsored, single-arm trial with short follow-up, and deliverability, repeated use within a single procedure, and performance in CTO lesions have not been systematically evaluated.

## 14. OCT in CTO PCI

Unlike IVUS, which can be used at any stage of the procedure, even within a vessel that is not yet patent, OCT requires that blood flow through the lesion has already been restored. Because the technique relies on light rather than ultrasound, image acquisition requires complete clearance of blood from the vessel lumen by forceful contrast or saline injection. This is possible only once antegrade flow has been successfully re-established. Importantly, the need for forceful injection itself carries clinical risk, as it may cause iatrogenic hydraulic dissection and expansion of an existing subintimal hematoma. In practice, this means that the use of OCT in CTO PCI is limited to the stage following successful lesion recanalization, and the technique cannot be used to guide the crossing procedure in real time, as is possible with IVUS [43].

In the ACE-CTO study, Sherbet et al. evaluated stent malapposition and strut coverage using OCT. They enrolled 100 patients undergoing CTO PCI, of whom 62 underwent follow-up OCT imaging in 8 months; a total of 44,450 struts across 6047 frames were analyzed, of which 9.3% were malapposed and 2.8% were uncovered. In total, 55 of 62 patients had at least one malapposed strut, and 50 had at least one uncovered strut [44]. The ALSTER-OCT-CTO registry showed similar findings: patients undergoing PCI for CTO lesions had higher rates of strut malapposition and delayed strut coverage than patients with non-CTO lesions at clinically driven angiographic follow-up 6.5 ± 2.1 months after PCI [45]. It should be noted that this use of OCT is not novel in CTO PCI but rather an extension of its established role in standard PCI. What is new is the observation that CTO lesions show higher rates of strut malapposition and incomplete strut coverage than non-CTO lesions.

## 15. Drug-Coated Balloons in CTO PCI

The use of drug-coated balloons (DCBs) in CTO PCI remains an actively investigated topic, with two distinct evidence bases depending on the lesion type.

### 15.1. DCB in In-Stent Restenosis

Evidence from randomized trials and meta-analyses shows that DCB treatment is effective in in-stent restenosis (ISR), with lower restenosis rates than plain old balloon angioplasty and outcomes that are comparable to, and in some trials less favorable than, repeat drug-eluting stent (DES) implantation; the benefit relative to DES therefore depends on the stent type and the length of follow-up [46,47]. Given the high risk of ISR and stent thrombosis (ST) in CTO lesions, it is important to determine whether DCBs are equally effective and safe in the de novo setting.

### 15.2. DCB in De Novo CTO Lesions

DCB-only treatment in de novo CTO lesions remains investigational and should not currently be considered equivalent to DES implantation in routine CTO PCI, particularly in lesions requiring extensive dissection or re-entry procedures. The AMAT registry will evaluate DCB use in de novo lesions, DCB combined with DES, and DCB in ISR.

Jun et al. retrospectively investigated 101 vessels with de novo CTO lesions treated with balloon angioplasty; 93 vessels successfully treated with a DCB-only strategy were analyzed; excluding the 8 remaining vessels introduces selection bias. No procedural complications occurred, and three vessels required bailout stenting. The median follow-up was 720 days. MACEs occurred in 8.3% of patients at 1 year, including cardiac death (1.2%) and target-vessel revascularization (TVR; 7.1%), with no non-fatal myocardial infarction or target-vessel thrombosis. At 2 years, MACEs occurred in 16.7% of patients, including cardiac death (2.4%), non-fatal myocardial infarction (3.6%), and TVR (13.1%), with no target-vessel thrombosis [48]. Because events were reported partly per patient and partly per vessel, these rates should be interpreted with care. These findings are encouraging and suggest that, when balloon angioplasty achieves an optimal result, a DCB-only strategy for de novo CTO may be effective and safe. However, this evidence comes from a retrospective study, and head-to-head comparisons of DCB and DES in CTO are needed.

Qin et al. compared DCB-only treatment in de novo CTO PCI with a hybrid approach combining DCB and DES. They enrolled 154 patients with de novo CTO lesions treated with DCB, including 57 in the hybrid group and 97 in the DCB-only group. During a median follow-up of 470 days, the incidences of target lesion revascularization (TLR), myocardial infarction, and cardiac death were 11.0%, 1.3%, and 1.9%, respectively, with no significant differences between the groups, and long-term TLR-free survival was also comparable. However, procedures in the hybrid group were more complex, as reflected by higher J-CTO scores, more frequent retrograde approaches, greater use of IVUS guidance and CTO guidewires, and longer procedural times [49].

It is worth noting that nearly all of the MACEs in the study by Jun et al. stem from the revascularization component (TVR, 13.1% at 2 years), whereas cardiac death (2.4%) and myocardial infarction (3.6%) remain low. Because the study had no DES comparator, these rates cannot be judged against DES outcomes, and a definitive assessment will require dedicated comparative trials. Results from larger multicenter trials and registries, including the CTO de novo registry and Piccolino, are awaited.

Overall, DCB-only treatment appears to be a promising option for less complex de novo CTO PCI because it may reduce the risk of ISR and ST. DCBs may also help address the problem of the downregulated, small distal vessel seen after successful CTO recanalization, as they allow the vessel to enlarge once antegrade flow is restored, potentially avoiding stent malapposition. Nevertheless, in CTO procedures involving extensive dissection, such as antegrade or retrograde dissection and re-entry, stent implantation may remain the only feasible option.

## 16. Practical Safety Considerations and Regulatory Status

Several of the techniques reviewed here are used at the edge of, or outside, manufacturer-labeled indications, and their safety profiles are not yet defined. Operators should keep the following points in mind:

- RODIN-CUT and ORCAS: the inflation pressures described (16–20 atm) may exceed the rated burst pressure of some cutting balloons. The instructions for use of the specific device should be checked before inflating above the labeled pressure because balloon rupture and vessel injury are possible.
- CBT: balloon remnants may become entrapped in calcified or tortuous vessels, and tamponade still occurred in a proportion of patients in the multicenter series; pericardiocentesis capability and a bailout plan are therefore essential.
- Condom technique: stent implantation leaves a permanent implant in the distal vessel, with implications for dual-antiplatelet therapy and long-term patency; precise sizing is required.
- MAT: an anchor balloon sized 1:1 to the vessel carries a risk of ischemia during prolonged inflation and of dissection or perforation if oversized; distal vessels in CTO may be downregulated, which complicates sizing.
- HDR and the Carlino technique: contrast injected through an extraplaque microcatheter may extend dissection planes, and registry data show higher perforation rates; injections should be small, slow, and visualized in orthogonal projections.
- Plasmawire: radiofrequency energy delivered near the vessel wall carries a theoretical risk of thermal injury and perforation, and safety has been reported only up to 30 days in a small first-in-human series.
- MHA: a 7 Fr forearm sheath carries a risk of radial artery occlusion and may be unsuitable in small radial arteries.

The regulatory status (CE mark, FDA clearance or approval) and the labeled indications of these technologies differ between regions and change over time. The peripheral Shockwave Javelin catheter is FDA-cleared, whereas the coronary version is investigational and under evaluation in the FORWARD CAD IDE study [40]. LithiX received CE mark in 2025 [42].

## 17. Limitations of This Review

This review has several limitations. First, it is a narrative review without a registered protocol, and no formal risk-of-bias assessment was performed, so study selection may be subject to selection bias. Second, the evidence for most techniques consists of case reports, small single-center series, and early-phase single-arm studies, and for some devices, it consists of manufacturer or trade-press material; conclusions on efficacy and safety are therefore tentative. Third, only English-language publications were included, and publication bias is likely because novel techniques are mainly reported by their developers. Fourth, the cut balloon technique was described by a member of the author team [14,15], and the authors’ own experience contributes to the practical commentary (Section 2.7); to limit the resulting risk of bias, own-experience statements are marked as such and sources are graded by evidence level (see the summary table in the Conclusions section). Finally, the field is evolving rapidly, and the evidence and regulatory status of the devices discussed may change after the search date (October 2026).

## 18. Conclusions

None of the rescue or plaque-modifying techniques described in this review can substitute for careful pre-procedural risk–benefit assessment. Preventing complications such as coronary artery perforation through appropriate patient selection, lesion assessment, and strategy choice remains more important than managing complications after they occur. CTO PCI remains one of the greatest challenges in modern interventional cardiology. Dedicated wires and emerging technologies may improve procedural safety and success rates in selected patients. Further evidence from registries and randomized trials is needed to better define patient benefit, refine indications, expand the appropriate use of CTO PCI, increase operator experience, and improve procedural efficiency. Despite the rapid development of new techniques and technologies, technical success rates in large registries have increased only moderately in recent years, and the field is still awaiting a true game changer.

Table 4 summarizes the proposed indications, evidence levels, study sizes, advantages, limitations, and current clinical status of the techniques and technologies reviewed.

## Figures and Tables

**Table 1:** Procedural comparison between the ORCAS and WALPO techniques.

Feature	ORCAS Technique	WALPO Technique
Primary mechanism	Real-time IVUS guidance	Use of an ostial protection guidewire
Plaque preparation	Integrated RODIN-CUT (sequential high-pressure cutting balloon inflations)	Focused primarily on stent positioning rather than on a specific preparation protocol
Visualization method	Direct, continuous visualization of anatomical landmarks (aortic cusp/ostium)	Mechanical/radiographic positioning using a specialized guidewire
Decision-making	Iterative process based on real-time feedback on plaque fragmentation	Static positioning based on the mechanical “stop” provided by the protective wire
Equipment requirements	Large-bore guide catheter (e.g., 7 Fr) to accommodate IVUS and therapeutic devices	Standard interventional equipment with a dedicated protection guidewire
Precision goal	Full ostial coverage with minimal aortic protrusion (<1 mm)	Avoidance of geographic miss through mechanical protection
Limitations	Large-bore access required to introduce IVUS	Less precise; possible geographic miss of the ostium in difficult cases

**Table 2:** CT-RECTOR score outcomes: relationship between the difficulty groups derived from the CT-RECTOR score and the likelihood of successful guidewire crossing within 30 min [35].

Difficulty Group	CT-RECTOR Score	No. of CTO Cases	Likelihood of Success Within 30 min (%)
Easy	0	22	95
Intermediate	1	60	88
Difficult	2	67	57
Very difficult	≥3	91	22

**Table 3:** Relationship between the difficulty groups derived from the J-CTO score and the likelihood of successful guidewire crossing within 30 min [37].

Difficulty Group	J-CTO Score	Cases, Derivation Set (*n* = 329)	Cases, Validation Set (*n* = 165)	Success Within 30 min, Derivation Set (%)	Success Within 30 min, Validation Set (%)
Easy	0	65	26	87.7	92.3
Intermediate	1	82	48	67.1	58.3
Difficult	2	92	46	42.2	34.8
Very difficult	≥3	90	45	10.0	22.2
Total		329	165	—	—

**Table 4:** Summary of novel techniques and emerging technologies in CTO PCI.

Technique/Device	Proposed Indication	Evidence Level	N (Patients/Lesions)	Major Advantages	Limitations	Current Clinical Status
MHA	Reducing vascular access-site complications in CTO PCI	Levels 2–3. Prospective (Agostoni) and retrospective (Zivelonghi) single-center studies	54 patients/56 procedures (prospective) + 91 (retrospective)	Smaller sheaths; avoid femoral access	Blind wiring increases perforation risk; requires experienced operators	High-volume referral centers
CBT	Sealing distal coronary perforation	Levels 3–4. Single-center case series + multicenter study	6 + 26	Inexpensive; simple; no microcatheter needed	Risk of balloon entrapment in calcified/tortuous vessels	Usable even in less experienced centers
Condom technique	Sealing distal coronary perforations	Level 4. Technical/innovation report	Not reported	Seals by stent implantation; inexpensive and adaptable (per technical report)	Requires precise balloon/stent sizing; permanent implant in the distal vessel; no outcome data	Early stage; technical-report-level evidence
TD-ADR	Precise true-lumen re-entry in ADR	Levels 3–4. Case reports (2) + retrospective four-center comparison with Stingray-ADR	1 + 1 + 27 (TD-ADR) vs. 27 (Stingray-ADR)	Precise re-entry direction; no Stingray needed	Technically demanding; time-consuming; needs a second operator	Small retrospective series and case reports
HDR	Crossing long, calcified lesions with ambiguous caps	Level 3. Single-center case series	43 patients/45 CTOs	100% procedural success in a single-center series by the developers; reported as reproducible	Small sample; possible selection bias	Preliminary data; confirmation in larger studies needed
MAT	Retrograde CTO PCI in post-CABG anatomy	Level 4. Case report	*n* = 1	Stabilizes the microcatheter; facilitates cap penetration	Risk of ischemia, and of perforation if sizing is misjudged	Case-report-level evidence only
RODIN-CUT	Fracturing severely calcified plaque under IVUS	Level 4 (technique itself); indirect evidence from non-CTO trials. Technical description + indirect evidence from unrelated RCTs (COPS, PREPARE-CALC-COMBO) on high-pressure cutting balloons in general	Not reported for RODIN-CUT itself	Confirmed cutting/chiseling effects on IVUS	Limited to larger vessels; fissure assessment harder with a second wire	Preliminary evidence; direct clinical data for the technique itself remain limited
ORCAS	Precise stenting of ostial lesions	Level 4. Case report	*n* = 1	Reduces reliance on angiography; aims at minimal aortic protrusion	Not a crossing technique; requires a 7 Fr catheter	Case-report-level evidence only
Plasmawire	Facilitating CTO crossing via RF energy	Level 4. First-in-human study (case series)	*n* = 7	Effective in antegrade and retrograde re-entry	Poorer results in heavy calcification; requires a 3D map	Early stage; coronary studies are just beginning
CTCA/CT-RECTOR	Pre-procedural planning; prediction of crossing success	Level 2. Multicenter registry (CT-RECTOR) + systematic review and meta-analysis (47 studies; Liang et al.)	240 lesions (CT-RECTOR)	Non-invasive; good agreement with J-CTO	No formal recommendation in revascularization guidelines	Growing use in referral centers; expert best-practice level
Shockwave Javelin	Calcium modification and crossing of calcified, difficult-to-cross lesions (coronary use investigational)	Level 4. Coronary first-in-human series; peripheral clinical use	20 (11 CTO)	Simultaneous modification and crossing; Javelin alone sufficient in 65%	Very small first-in-human series; 35% needed additional balloon-based IVL; no CTO-specific outcomes; pivotal data pending	Investigational in coronary arteries (FORWARD CAD IDE); peripheral version FDA-cleared
Hertz Contact IVL (LithiX)	Treatment of calcified coronary arteries	Level 2. Prospective single-arm multicenter trial (PINNACLE-I), peer-reviewed; industry-sponsored	60 patients/63 lesions	High clinical and angiographic success; good safety profile	Single-arm trial; short-term (6-month) follow-up; CTO-specific data not reported	[CE-marked (2025)]; needs confirmation in larger populations
OCT in CTO	Post-implantation assessment (malapposition, strut coverage)	Level 2. Prospective study (ACE-CTO) + registry (ALSTER-OCT-CTO)	100 (ACE-CTO); not reported (ALSTER-OCT-CTO)	Detects higher rates of abnormalities than in non-CTO lesions	Cannot guide real-time crossing	Established role from standard PCI; not novel for CTO
DCB in CTO	(a) ISR: established; (b) de novo: investigational	(a) Levels 1–2; (b) Level 3. (a) Multiple studies/meta-analyses; (b) 2 retrospective studies	(b) 93 (Jun) + 154 (Qin)	May reduce restenosis/thrombosis without a permanent implant	For de novo CTO: retrospective data only; no head-to-head RCT vs. DES	Established for ISR; investigational for de novo CTO

## Data Availability

No new datasets were generated or analyzed. The illustrations accompanying the technique descriptions in Section 4, Section 5, Section 6 and Section 7 are illustrative images from the authors’ own clinical and bench practice (see Section 2.7).

## Abbreviations

The following abbreviations are used in this manuscript:

ADR	antegrade dissection and re-entry
AW	antegrade wiring
AWE	antegrade wire escalation
CABG	coronary artery bypass grafting
CB	cutting balloon
CBT	cut balloon technique
CT	computed tomography
CTCA	CT coronary angiography
CT-RECTOR	Computed Tomography Registry of Chronic Total Occlusion Revascularization
CTO	chronic total occlusion
DCB	drug-coated balloon
DES	drug-eluting stent
EACVI	European Association of Cardiovascular Imaging
EAPCI	European Association of Percutaneous Cardiovascular Interventions
ESC	European Society of Cardiology
HC IVL	Hertz Contact intravascular lithotripsy
HDR	HydroDynamic contrast recanalization
HFmrEF	heart failure with mildly reduced ejection fraction
HFrEF	heart failure with reduced ejection fraction
ISR	in-stent restenosis
IVL	intravascular lithotripsy
IVUS	intravascular ultrasound
J-CTO	Multicenter CTO Registry in Japan (score)
LAST	limited antegrade subintimal tracking
MACCE	major adverse cardiac and cerebrovascular events
MACE	major adverse cardiovascular events
MAT	microcatheter anchor technique
MHA	minimalistic hybrid approach
MLA	minimal lumen area
MSA	minimal stent area
OA	orbital atherectomy
OCT	optical coherence tomography
ORCAS	Ostial RODIN-CUT Angioplasty and Stenting
PCI	percutaneous coronary intervention
PTA	percutaneous transluminal angioplasty
RA	rotational atherectomy
RDR	retrograde dissection and re-entry
RF	radiofrequency
RODIN-CUT	technique name (not a formal acronym) for sequential cutting balloon inflations under real-time IVUS guidance
RW	retrograde wiring
RWE	retrograde wire escalation
SHP	super-high-pressure (balloon)
ST	stent thrombosis
STAR	subintimal tracking and re-entry
TD-ADR	tip-detection antegrade dissection and re-entry
TIMI	Thrombolysis in Myocardial Infarction
TLR	target lesion revascularization
TVR	target vessel revascularization
WALPO	Wire in Aorta for Localization and Protection of the Ostium

## References

[1] Werner G.S., Martin-Yuste V., Hildick-Smith D., Boudou N., Sianos G., Gelev V., Rumoroso J.R., Erglis A., Christiansen E.H., Escaned J. (2018). A Randomized Multicentre Trial to Compare Revascularization with Optimal Medical Therapy for the Treatment of Chronic Total Coronary Occlusions. Eur. Heart J..

[2] Lee S.-W., Lee P.H., Ahn J.-M., Park D.-W., Yun S.-C., Han S., Kang H., Kang S.-J., Kim Y.-H., Lee C.W. (2019). Randomized Trial Evaluating Percutaneous Coronary Intervention for the Treatment of Chronic Total Occlusion: The DECISION-CTO Trial. Circulation.

[3] Behnes M., Schmidberger M., Mashayekhi K., Vadalà G., Moroni A., Gutierrez-Chico J.L., Ayoub M., Christiansen E.H., Pyxaras S., Bufe A. (2025). Coronary chronic total occlusions affect long-term prognosis in heart failure with mildly reduced ejection fraction. J. Am. Heart Assoc..

[4] Megaly M., Zakhour S., Maki M., Albusoul L., Nakhle A., Karacsonyi J., Mashayekhi K., Rinfret S., Brilakis E.S., Alaswad K. (2025). Impact of chronic total occlusion PCI in non-LAD coronary arteries on patients with cardiomyopathy. Am. J. Cardiol..

[5] Kostantinis S., Simsek B., Karacsonyi J., Alaswad K., Krestyaninov O., Khelimskii D., Karmpaliotis D., Jaffer F.A., Khatri J.J., Poommipanit P. (2022). In-hospital outcomes and temporal trends of percutaneous coronary interventions for chronic total occlusion. EuroIntervention.

[6] Vadalà G., Galassi A.R., Werner G.S., Sianos G., Boudou N., Garbo R., Maniscalco L., Bufe A., Avran A., Gasparini G.L. (2024). Contemporary outcomes of chronic total occlusion percutaneous coronary intervention in Europe: The ERCTO registry. EuroIntervention.

[7] Sapontis J., Salisbury A.C., Yeh R.W., Cohen D.J., Hirai T., Lombardi W., McCabe J.M., Karmpaliotis D., Moses J., Nicholson W.J. (2017). Early procedural and health status outcomes after chronic total occlusion angioplasty: A report from the OPEN-CTO registry. JACC Cardiovasc. Interv..

[8] Vescovo G.M., Zivelonghi C., Scott B., Wilgenhof A., Willemen Y., Convens C., Verheye S., Vermeersch P., Agostoni P. (2021). A new approach to percutaneously treat chronic coronary total occlusions: The “minimalistic hybrid approach” algorithm. EMJ Int. Cardiol..

[9] Agostoni P., Scott B., Budassi S., Verheye S., Convens C., Vermeersch P., Zivelonghi C. (2021). A prospective evaluation of a redefined version of the “minimalistic hybrid approach” algorithm for percutaneous coronary chronic total occlusion revascularization. Catheter. Cardiovasc. Interv..

[10] Zivelonghi C., van Kuijk J.P., Poletti E., Suttorp M.J., Eefting F.D., Rensing B.J., Ten Berg J.M., Colombo A., Azzalini L., Agostoni P. (2020). A “minimalistic hybrid algorithm” in coronary chronic total occlusion revascularization: Procedural and clinical outcomes. Catheter. Cardiovasc. Interv..

[11] Abdalwahab A., Farag M., Brilakis E.S., Galassi A.R., Egred M. (2021). Management of coronary artery perforation. Cardiovasc. Revasc. Med..

[12] He L.Y., Han J.L., Guo L.J., Zhang F.C., Cui M., Gao W. (2015). Effect of transcatheter embolization by autologous fat particles in the treatment of coronary artery perforation during percutaneous coronary intervention. Chin. Med. J..

[13] Tanaka S., Nishigaki K., Ojio S., Yasuda S., Okubo M., Yamaki T., Kubota T., Takasugi N., Ishihara Y., Kawasaki M. (2008). Transcatheter embolization by autologous blood clot is useful management for small side branch perforation due to percutaneous coronary intervention guide wire. J. Cardiol..

[14] Sobieszek G., Zięba B. (2020). Balloon fragment technique used to close distal coronary vessel perforation. J. Invasive Cardiol..

[15] Sobieszek G., Zięba B., Dworzański W., Celiński R., Barbero U., Opolski M.P. (2023). Embolization of perforated coronary artery with a fragment of balloon catheter (cut balloon technique): Multicenter study. J. Cardiovasc. Dev. Dis..

[16] Agostoni P., Moroni A., Scott B., Poletti E., Benedetti A., Zivelonghi C. (2024). An adaptable, cheap and easy-to-use interventional tool to obtain vascular sealing: The condom technique. JACC Cardiovasc. Interv..

[17] Suzuki S., Okamura A., Nagai H., Iwakura K. (2022). Tip detection-antegrade dissection and reentry using intravascular ultrasound in chronic total occlusion intervention: First human case report. Eur. Heart J. Case Rep..

[18] Tadano Y., Kuramitsu S., Sugie T., Kanno D., Fujita T. (2024). Tip detection-antegrade dissection and re-entry (TD-ADR) with integrated fluoroscopic and intravascular ultrasound images in chronic total occlusion: First case report of integrated TD-ADR technique. Eur. Heart J. Case Rep..

[19] Tanaka K., Okamura A., Yoshikawa R., Tsuchikane E., Ishikawa M., Suzuki S., Nagai H., Sumiyoshi A., Kawahira M., Yamasaki T. (2024). Tip Detection–Antegrade Dissection and Re-Entry with New Puncture Wire in CTO Intervention: Revolution Through 3D-Wiring. JACC Asia.

[20] Carlino M., Azzalini L. (2023). What is the Carlino technique?. EuroIntervention.

[21] Carlino M., Ruparelia N., Thomas G., Brooks M., Uretsky B.F., Brilakis E.S., Karmpaliotis D., Hanratty C., Walsh S., Spratt J. (2016). Modified contrast microinjection technique to facilitate chronic total occlusion recanalization. Catheter. Cardiovasc. Interv..

[22] Alexandrou M., Rempakos A., Al Ogaili A., Choi J.W., Poommipanit P., Khatri J., Elbarouni B., Love M.P., Jaber W., Rinfret S. (2023). Use of the Carlino Technique in Chronic Total Occlusion Percutaneous Coronary Intervention. Am. J. Cardiol..

[23] Carlino M., Nascimbene A., Brilakis E.S., Yarrabothula A., Colombo A., Nakamura S., Azzalini L., Hanif B., Iqbal M.B., Arain S.A. (2024). HydroDynamic contrast recanalization (HDR): Description of a new crossing technique for coronary chronic total occlusions. Catheter. Cardiovasc. Interv..

[24] Budassi S., Zivelonghi C., Dens J., Bagnall A.J., Knaapen P., Avran A., Spratt J.C., Walsh S., Faurie B., Agostoni P. (2021). Impact of prior coronary artery bypass grafting in patients undergoing chronic total occlusion-percutaneous coronary intervention: Procedural and clinical outcomes from the RECHARGE registry. Catheter. Cardiovasc. Interv..

[25] Iqbal M.B., Vo M., Vijayaraghavan R., Faurie B., Leibundgut G., Carlino M., Rinfret S., Brilakis E.S. (2024). A microcatheter anchor technique to prevent prolapse and facilitate retrograde chronic total occlusion intervention via a retroflexed saphenous vein graft. Cardiovasc. Revasc. Med. Interes. Cases.

[26] Ungureanu C., Leibundgut G., Cocoi M., Gasparini G., Colletti G., Avran A., Poletti E., Moroni A., Mangieri A., Dumitrascu S. (2025). Enhanced efficiency of sequential cutting balloon angioplasty in calcified coronary artery disease: The RODIN-CUT technique. Catheter. Cardiovasc. Interv..

[27] Mangieri A., Nerla R., Castriota F., Reimers B., Regazzoli D., Leone P.P., Gasparini G.L., Khokhar A.A., Laricchia A., Giannini F. (2023). Cutting balloon to optimize predilation for stent implantation: The COPS randomized trial. Catheter. Cardiovasc. Interv..

[28] Allali A., Toelg R., Abdel-Wahab M., Hemetsberger R., Kastrati A., Mankerious N., Traboulsi H., Elbasha K., Rheude T., Landt M. (2022). Combined rotational atherectomy and cutting balloon angioplasty prior to drug-eluting stent implantation in severely calcified coronary lesions: The PREPARE-CALC-COMBO study. Catheter. Cardiovasc. Interv..

[29] Sharma S.K., Mehran R., Vogel B., Hooda A., Sartori S., Hanstein R., Feng Y., Shlofmitz R.A., Jeremias A., Spirito A. (2024). Rotational Atherectomy Combined with Cutting Balloon to Optimise Stent Expansion in Calcified Lesions: The ROTA-CUT Randomised Trial. EuroIntervention.

[30] Ungureanu C., Cocoi M., Boukhris M., Colletti G., Jossart A., Trefois Q., Leibundgut G. (2025). IVUS-guided ostial lesion preparation with sequential cutting balloon inflations and stent placement: The ORCAS technique. Clin. Case Rep..

[31] Leibundgut G., Kovacic M., Cocoi M., Rinfret S. (2024). Interventional applications for an ostial protection guidewire: The WALPO technique. Catheter. Cardiovasc. Interv..

[32] Kanno D., Tsuchikane E., Nasu K., Katoh O., Kashima Y., Kaneko U., Fujita T., Suzuki Y., Suzuki T. (2018). Initial results of a first-in-human study on the PlasmaWire system, a new radiofrequency wire for recanalization of chronic total occlusions. Catheter. Cardiovasc. Interv..

[33] Yassin F., Khan J., Mozid A., Connolly D., Sharma V. (2023). The utility of CT coronary angiography in chronic total occlusion percutaneous coronary intervention. Eur. Cardiol..

[34] Opolski M.P., Achenbach S. (2015). CT angiography for revascularization of CTO: Crossing the borders of diagnosis and treatment. JACC Cardiovasc. Imaging.

[35] Opolski M.P., Achenbach S., Schuhbäck A., Rolf A., Möllmann H., Nef H., Rixe J., Renker M., Witkowski A., Kepka C. (2015). Coronary computed tomographic prediction rule for time-efficient guidewire crossing through chronic total occlusion: Insights from the CT-RECTOR multicenter registry. JACC Cardiovasc. Interv..

[36] Liang S., Bai Y., Zhang J., Wang A., Li J., Diao K., He Y. (2024). The added value of coronary CTA in chronic total occlusion percutaneous coronary intervention: A systematic review and meta-analysis. Eur. Radiol..

[37] Morino Y., Abe M., Morimoto T., Kimura T., Hayashi Y., Muramatsu T., Ochiai M., Noguchi Y., Kato K., Shibata Y. (2011). Predicting successful guidewire crossing through chronic total occlusion of native coronary lesions within 30 minutes: The J-CTO score as a difficulty grading and time assessment tool. JACC Cardiovasc. Interv..

[38] Galassi A.R., Vadalà G., Werner G.S., Cosyns B., Sianos G., Hill J., Dudek D., Picano E., Novo G., Andreini D. (2024). Evaluation and management of patients with coronary chronic total occlusions considered for revascularisation. A clinical consensus statement of EAPCI, EACVI, and the ESC Working Group on Cardiovascular Surgery. EuroIntervention.

[39] Corl J.D., Paprzycki C. (2025). The Shockwave Javelin Peripheral Intravascular Lithotripsy Catheter. Endovascular Today.

[40] Strange J.W., Gallagher S., Wilson S.J., Malhotra G., Li C., Sidik N., Johnson M., West N.E.J., Spratt J.C. (2025). First-in-Human Study of a Novel Coronary Crossing Device for Calcified Lesions. JACC Cardiovasc. Interv..

[41] Bennett J., Hamer B., Paradies V., Tonino P., Bataille Y., Verheye S. (2024). TCT-381 Safety and effectiveness of a novel intravascular lithotripsy device using the Hertz contact stress mechanism for calcium fragmentation: Six-month outcomes of the PINNACLE I clinical trial. J. Am. Coll. Cardiol..

[42] Verheye S., Hamer B., Paradies V., Tonino P., Bataille Y., Bennett J. (2024). TCT-383 Mechanistic effects of coronary Hertz contact intravascular lithotripsy on treatment of calcified lesions: PINNACLE I OCT substudy procedural imaging outcomes. J. Am. Coll. Cardiol..

[43] Xenogiannis I., Pavlidis A.N., Kaier T.E., Rigopoulos A.G., Karamasis G.V., Triantafyllis A.S., Vardas P., Brilakis E.S., Kalogeropoulos A.S. (2023). The role of intravascular imaging in chronic total occlusion percutaneous coronary intervention. Front. Cardiovasc. Med..

[44] Sherbet D.P., Christopoulos G., Karatasakis A., Danek B.A., Kotsia A., Navara R., Michael T.T., Roesle M., Rangan B.V., Haagen D. (2016). Optical coherence tomography findings after chronic total occlusion interventions: Insights from the ACE-CTO study. Cardiovasc. Revasc Med..

[45] Heeger C.H., Busjahn A., Hildebrand L., Fenski M., Lesche F., Meincke F., Kuck K.H., Bergmann M.W. (2016). Delayed coverage of drug-eluting stents after interventional revascularisation of chronic total occlusions assessed by optical coherence tomography: The ALSTER-OCT-CTO registry. EuroIntervention.

[46] Giacoppo D., Alfonso F., Xu B., Claessen B.E., Adriaenssens T., Jensen C., Pérez-Vizcayno M.J., Kang D.Y., Degenhardt R., Pleva L. (2020). Drug-coated balloon angioplasty vs. drug-eluting stent implantation in patients with coronary stent restenosis. J. Am. Coll. Cardiol..

[47] Scheller B., Hehrlein C., Bocksch W., Rutsch W., Haghi D., Dietz U., Böhm M., Speck U. (2006). Treatment of coronary in-stent restenosis with a paclitaxel-coated balloon catheter. N. Engl. J. Med..

[48] Jun E.J., Shin E.S., Teoh E.V., Bhak Y., Yuan S.L., Chu C.M., Garg S., Liew H.B. (2022). Clinical outcomes of drug-coated balloon treatment after successful revascularization of de novo chronic total occlusions. Front. Cardiovasc. Med..

[49] Qin Q., Chen L., Ge L., Qian J., Ma J., Ge J. (2023). Long-term clinical outcomes of drug-coated balloon for the management of chronic total occlusions. Coron. Artery Dis..

